# The practical value of frost-tolerance markers in winter durum wheat breeding

**DOI:** 10.3389/fpls.2026.1959465

**Published:** 2026-10-21

**Authors:** Mikhail G. Divashuk, Aleksey S. Yanovsky, Varvara A. Gorskaia, Alexandra A. Mudrova, Maria S. Esina, Ludmila A. Bespalova, Viktoria S. Voronezhskaya, Mikhail G. Monakhos, Anastasiya D. Voropaeva, Anastasiya G. Chernook, Andrey V. Arkhipov, Daniil S. Ulyanov, Gennady I. Karlov

**Affiliations:** 1 All-Russian Research Institute of Agricultural Biotechnology, Moscow, Russia; 2 P. P. Lukyanenko National Grain Center, Krasnodar, Russia

**Keywords:** copy number variation, Fr-A2, frost tolerance, heading date, marker-assisted selection, non-Fr loci, *Triticum durum*, Vrn-A1

## Abstract

Frost tolerance limits the expansion of winter durum wheat (*Triticum durum*) into continental zones. Its control is attributed mainly to chromosome 5A loci, *Fr-A2* [the *CBF* cluster: *CBF-A14* copy number variation (CNV) and a non-CNV component], and *Fr-A1*/*VRN-A1*. We assessed candidate KASP (kompetitive allele-specific polymerase chain reaction) markers in the Russian winter durum wheat gene pool (161 accessions) and in a segregating F_2_ panel from crosses with foreign frost-susceptible lines. Frost tolerance was measured as the percentage of plants surviving a controlled freezing test. In the collection, the favorable *Fr-A2* class is nearly fixed at all four markers of the *CBF-A14* copy-number haplotype [minor allele frequency (MAF), 0.04 to 0.08]. Minor-class lines survive at 7.3% to 13.5% against 43.7% to 45.4%, but only 2 of the 15 come from the Krasnodar program, a contrast confounded with origin. The frequency of the favorable class rose from 0.60 to 0.87 among accessions entered into State Variety Trials before 2000 to 0.95 to 1.00 after 2010. The non-CNV component S1051014 is polymorphic and not associated with the trait (*p* = 0.442). In the F_2_ panel, the dose of the adapted-parent allele among survivors rose from 1.510 to 1.750 at S2269949 (within-family randomization test, one-sided *p* = 0.005, two-sided *p* = 0.010) and from 1.752 to 1.958 at S1077313 (*p* = 0.038 and 0.076). Of three polymorphic independent non-*Fr* loci, 7B (Mor866) showed no effect, whereas 1B (Mor587, allele T) and 5B (Mor534, allele G) showed a nominal effect of the same direction, approximately 7 to 8 percentage points (*p* = 0.048 and 0.052); the long-vernalization *VRN-A1* allele was associated with higher survival (*p* = 0.019). These markers, untested in segregating material, are presented as candidates. Later heading accompanied higher survival (+0.0142 per day on the angular scale, *p* = 0.0004, *n* = 155), largely a between-program contrast. The *Fr-A2* markers are the most valuable when external frost-susceptible material is used. With *Fr-A2* fixed, the choice of marker follows from the crossing plan rather than from the published effect size.

## 1 Introduction

Frost tolerance determines the overwintering of winter wheat and, ultimately, the limits of its cultivation. For durum wheat (*Triticum durum* Desf.; a tetraploid with the A and B genomes), it is especially critical. Durum is more sensitive to frost than bread wheat (*T. aestivum*), and this difference is the main constraint on its northward expansion (Longin et al., 2013; Sieber et al., 2016). The lack of frost tolerance long held back its spread into continental zones with severe winters, where autumn sowing brings gains in water supply and grain quality (Habib et al., 2026; Sieber et al., 2016). In autumn, during cold acclimation, the plant slows its growth, accumulates soluble sugars and osmoprotectants, remodels membrane lipids, and synthesizes cold-induced protective proteins (Hassan et al., 2021). The protective role of sugars during ice formation was argued by Maksimov (1913), the two-phase theory of hardening was developed by Tumanov (1979), and a modern synthesis of the role of sugars and protective proteins was given by Trunova (2007). Frost tolerance is a strongly polygenic trait. For breeding, it is therefore important to know how much of its genetic potential has already been realized through selection and how much remains available.

The pace of plant development is coupled to cold adaptation through the vernalization gene *VRN-A1*. Growth-habit alleles are defined by structural rearrangements of the promoter and the first intron (Fu et al., 2005; Yan et al., 2003, Yan et al., 2004), while *VRN1* itself triggers the transition to flowering after cold exposure (Trevaskis, 2010). On chromosome 5A, the frost-tolerance locus *Fr-A1* is tightly linked to *VRN-A1*: the two map to the same region and have not been separated by recombination, but they are not the same gene. The second 5A locus, *Fr-A2*, has a larger effect. It is a cluster of *CBF* genes (C-repeat binding factors), first described in diploid *Triticum monococcum* (Miller et al., 2006). In cultivated wheat, its contribution combines the copy number variation (CNV) of the *CBF-A14* gene with a separate non-CNV component (Sieber et al., 2016; Würschum et al., 2017). In durum wheat, *CBF-A14* copy number has been shown to set the level of frost tolerance (Sieber et al., 2016). Functional KASP (kompetitive allele-specific polymerase chain reaction) markers suitable for routine selection have been developed for both loci (Rasheed et al., 2016; Sieber et al., 2016; Würschum et al., 2017), and *VRN-A1* haplotypes are described within a unified nomenclature based on diagnostic exons (Giménez et al., 2025). The markers, and the mechanisms behind them, have been studied mostly in bread wheat; thus, transfer to durum, which lacks the D genome, requires direct verification. The two species do differ at the genome level in ways that markers detect directly: genome-specific polymorphisms, among them a D-genome-linked insertion in the intergenic spacer, separate hexaploid from tetraploid wheat (Kasymova and Ulug, 2026).

The polygenic basis of frost tolerance extends beyond the *Fr* group and is clearly seen in related genomes. In rye (*Secale cereale*), the most frost-tolerant of the small-grain cereals, tolerance is inherited polygenically and rests on a set of *CBF*/*ICE1*/*COR* genes (Li et al., 2011). Triticale (× *Triticosecale*) inherits wheat (5A and 1B) and rye (5R) components (Liu et al., 2014). In bread wheat, frost tolerance maps as a polygenic trait with contributions outside *Fr* (Soleimani et al., 2022). Non-*Fr* determinants are expected in durum as well: loci on 1B, 5B, and 7B, for which KASP markers have been developed, were studied in material obtained within the breeding program of the P.P. Lukyanenko National Grain Center (Divashuk et al., 2026; see also Lei et al., 2023, on cold-tolerance genes outside 5A).

Russian winter durum wheat is convenient for such an assessment: the informativeness of the published markers can be tested within a single adapted gene pool, in contrast to the pan-European panels of Sieber et al. (2016) and Habib et al. (2026). This breeding direction has been developed under continental conditions by the Krasnodar school of breeding (the Lukyanenko school), which has conducted directed selection for winter hardiness over several decades (Puchkov and Nabokov, 2001). The collection studied covers almost the entire current cultivar pool of Russian winter durum wheat, that is, all cultivated varieties and elite lines.

This work pursued two groups of aims. The first concerns the collection: (i) to determine, in the Russian winter durum wheat gene pool, the informativeness of the candidate 5A frost-tolerance markers (*Fr-A2*: the *CBF-A14* copy-number haplotype and the non-CNV component; *Fr-A1*/*VRN-A1*); (ii) to evaluate, for the first time in the same gene pool, the polymorphism and frost-tolerance association of three independent non-*Fr* loci (1B, 5B, and 7B); and (iii) to describe the association of frost tolerance with developmental timing. The second concerns segregating material: (iv) to test, in an F_2_ panel, the segregation of the *Fr-A2* markers and their association with survival under freezing.

## 2 Materials and methods

### 2.1 Plant material

Two sets of material were studied:

1. The collection: a near-complete (exhaustive) sampling of the modern Russian and Eastern European winter durum cultivar pool (*T. durum* Desf.), covering all cultivated varieties and elite lines (161 accessions with genotypic and phenotypic data; originator, country, and year of entry into State Variety Trials in **Supplementary Table S4**). The collection includes cultivars developed throughout the entire history of winter durum wheat breeding in Russia, not only recent releases. Work with durum wheat at the P.P. Lukyanenko National Grain Center dates from 1936, the year of the first report on durum wheat by P.P. Lukyanenko at the reporting conference of the institute (L.A. Bespalova, personal communication).

2. Segregating F_2_ populations (the second generation from a cross) served for a direct test of the association of *Fr-A2* markers with frost tolerance. This test cannot be performed on the collection itself. In our work, the *Fr-A2* markers proved nearly monomorphic in it (the favorable allele is fixed, Section 3.2); thus, the allele–trait association cannot be tested statistically within the collection. Segregating material fills this gap, because meiosis reshuffles genetic backgrounds and enrichment of the favorable allele among survivors directly indicates the action of the locus. We used F_2_ populations then present in the breeding process at the P.P. Lukyanenko National Grain Center. The adapted parents were the frost-tolerant cultivars Tsel and Sinora, crossed with foreign lines contrasting at *Fr-A2* (Ghibli, GK Julidur, Tito Flavio, Akhenaton, Massimo Meridio, and L2870) and with the adapted cultivars Odari, Andromeda, and Zernograd 333-17. The parental combination of every family was recovered from the primary breeding records and is given in **Supplementary Table S6**. Seven of the eight families are the F_2_ of a three-way cross (A/B//C) in which two parents are adapted and one is foreign. Only family 3320–3 is a simple two-parent F_2_. This determines the expected segregation ratio 5:2:1 in a three-way cross and 1:2:1 in a two-parent cross and, therefore, an expected allele dose of 1.50 in seven of the eight families. The cultivars Krupinka and Krucha served as frost-tolerance standards. Individual data for the eight families (plant, family, cross, status, and *Fr-A2* genotypes) are given in **Supplementary Table S3**.

The F_2_ experiment has the structure of a single population sampled twice. In the first season, 152 plants from eight families were genotyped before freezing. The plants were then frozen, and in the following season, the 52 plants that had survived were genotyped again. The survivors are a subset of the plants genotyped before freezing and are not an independent comparison group. Individual plant identity could not be carried through the freezing test with confidence, so no analysis in this work uses it. Family membership is certain, because families were kept in separate boxes, and the analysis conditions on family alone (Section 2.7). The denominator available for the F_2_ is the number of genotyped plants, since the number of plants that emerged before freezing was not retained in the analysis records.

### 2.2 Field and rack trials, heading date

Accessions were grown in a collection nursery on 5-m² plots in two replicates (**Supplementary Figure S4**) and on concrete racks at the experimental station of the P.P. Lukyanenko National Grain Center (Krasnodar) (**Supplementary Figure S3**), where overwintering was scored visually each year. The score is a 10-point scale from 0 to 9, from the lowest to the highest value, where 0 denotes complete absence of regrowth and 9 denotes maximum regrowth. To make scores comparable between years, each year is calibrated against the standards Krupinka and Donchanka, which are assigned a score of 2 by definition, and all other entries are scored relative to them. On the racks, sowing was to a depth of 5 cm, and snow was swept off the racks and the field plot through the winter. Overwintering is thereby assessed under conditions more demanding in terms of soil freezing, because the decisive factor becomes the soil temperature at the depth of the tillering node, which is markedly lower than under natural field conditions. Heading date was recorded in the field nurseries of the studied collection (2017–2023) and expressed as day of year (DOY). The per-accession mean heading dates are given in **Supplementary Table S11**. Heading was analyzed as an independent field developmental trait.

Within a year, accessions were assigned to boxes without reference to their expected performance. Across years, progression to a harsher regime was conditioned on earlier performance. An accession that failed at the −12 °C series was not normally carried forward to −16 or −17°C in a later year, and the harsher regimes were reserved for material that had already shown itself worth testing, together with the standards. Accessions subsequently tested at −16 or −17 °C had a mean survival of 51.8% in the −12 °C series against 33.2% for those that were not (Mann–Whitney *p* = 5.5 × 10^−5^, *n* = 25 and 77). The selection rule operated on the phenotype and not on any marker, and no marker was known for any accession at the time of allocation; thus, range restriction of this kind can attenuate a marker association within the harsher environments and cannot create one. A sensitivity analysis is reported in **Supplementary Table S8**.

### 2.3 Controlled freezing test

This section describes the freezing test of the collection; the F_2_ families were grown in the same type of boxes, one family per box, and were hardened and frozen under the same protocol, one regime per box (Section 2.1). Frost tolerance was assessed by a controlled freezing test in C-816 freezing chambers, following the standard protocol of the experimental station developed by the Krasnodar school of breeding for winter hardiness (Puchkov and Nabokov, 2001). Plants were hardened under natural autumn conditions in Krasnodar.

Plants were grown in wooden boxes 38 × 26 × 12 cm filled with a soil–sand mixture (3:1). Dry seeds were sown at the end of the locally optimal window (the second half of October), in seven rows 5 cm apart, 25 seeds per row, to a depth of 2 cm. Row 4 of each box was sown with the standard, cultivar Krupinka, whose frost-tolerance level is typical of winter durum wheat and which retains more than 50% of the plants in its row at −15 °C. The six test accessions were sown in randomized order in the remaining rows. One box was used per accession per regime. A box was rejected if the standard died at a temperature at which it should have survived. After natural hardening (November to December), the boxes were moved into the chambers. At above-zero temperatures, an additional hardening of 1 day at −2 and −5 °C was applied before loading. The boxes were placed on trolleys in three tiers. Box and tier identifiers were not retained in the analysis dataset and therefore do not enter the models (Section 2.7).

From −5 °C, the chamber temperature was lowered to the target at 1 °C per hour, held at the target for 24 h, and then raised at 1 °C per hour to +5 and then to +10 °C. The boxes were kept for 2 days at +10 °C in the chamber and were then moved to the glasshouse for 2 weeks at +25 °C, with a photoperiod of 10 h light in the first week and 14 h light in the second (**Supplementary Figure S5**). The thawing and recovery regime given here refers to the collection trial and differed from that applied to the F_2_ panel of Divashuk et al. (2026), a separate experiment in the same chambers. The standard series was set at −12 °C. More severe regimes of −14, −15, −16, and −17 °C were also used. The set of temperatures was chosen to span the whole frost-tolerance range of the collection. At loading, the plants were at the tillering stage. The growth stage was recorded descriptively and was not scored on the Zadoks scale. The final proportion of survivors depended strongly on the combination of year and regime and varied over a wide range (**Table 1**), which dictated the choice of mixed models with explicit accounting for the environment (Section 2.7).

**Table 1:** Frost tolerance (% survivors) of the studied collection by freezing regime or series and year (*n* is the number of accessions in the environment; *n* = 161).

Regime/series	Year	*n*	Mean survival, %
−12°C	2021	102	49.7
−12°C	2022	98	23.5
−12 °C	2023	98	38.6
−14 °C	2022	42	45.8
−15 °C	2020	77	72.8
−15 °C	2021	78	68.9
−15 °C	2022	55	35.7
−15 °C	2023	23	62.7
−16 °C	2020	47	45.7
−16 °C	2021	83	54.4
−16 °C	2022	40	4.3
−17°C	2021	78	41.4

The repeatability of a single freezing test, estimated from the variance components of the primary model, was *R* = 0.45 (between-accession variance 0.0357, residual variance 0.0433; **Supplementary Table S9**). With a harmonic mean of 4.1 environments per accession, the repeatability of the accession mean was *R* = 0.77. Year and regime account for 39.6% of the total variance of the angular response, and adding the accession factor brings the explained fraction to 71.2%. About 3 environments per accession are needed to reach *R* = 0.7, about 5 to reach *R* = 0.8, and about 11 to reach *R* = 0.9, which sets the replication required before lines are ranked on this trait. We report repeatability rather than heritability, because the collection is a set of fixed cultivars and elite lines and not a designed genetic population.

### 2.4 Frost-tolerance phenotyping

The primary trait of this study is the proportion of surviving plants in the row, expressed as a percentage of the plants present before freezing. It is the response variable in every model reported below. Seven to nine days after freezing, regrowth was scored visually on the same 10-point scale (0 to 9, **Supplementary Figure S1**), where 0 denotes complete absence of regrowth, that is, death of the aboveground biomass of the row, and 9 denotes maximum regrowth. This score is an operational measure used to decide which boxes to keep for the full recovery period, and it enters no model in this work. The two measures are correlated and are not interchangeable (Spearman rho = 0.29, *n* = 200 rows, *p* = 3.9 × 10^−5^). Surviving plants were counted at the end of the 2-week recovery period (**Supplementary Figure S2**). Three traits were recorded: frost tolerance (the proportion of surviving plants in the row, a quantitative measure), spring regrowth, and overwintering (scores, ordinal measures). Scores for regrowth and overwintering were on average high in the collection.

### 2.5 DNA extraction

Genomic DNA was extracted from young leaves separately for (i) accessions of the studied collection and (ii) F_2_ plants, on an Auto-Pure 96 automated station (Allsheng, China) with the MagnoPrime^®^ GMO kit (NextBio LLC, Russia) following the manufacturer’s protocol. DNA quality and concentration were checked on a SPECTROstar^®^ Nano spectrophotometer (BMG LABTECH, Germany); the extracted DNA was used as template for genotyping (Section 2.6).

### 2.6 Genotyping

Genotyping was carried out using KASP assays, which score single-nucleotide polymorphisms (SNPs). The Vrn-B1 marker was typed by polymerase chain reaction (PCR) followed by electrophoresis (see below). The signal of each sample was assigned to an allele class by fluorophore (FAM or HEX homozygote). The two materials were genotyped with different panels. The collection (161 accessions) was genotyped with the 11 markers of **Table 2**. The F_2_ panel (152 plants) was genotyped at *Fr-A2* with S2269949 and S1077313, because the F_2_ experiment was designed to test that locus. No non-*Fr* marker was applied to the F_2_ material; S1862541 and S1298957 were applied to the collection only, so the F_2_ result rests on two of the four *Fr-A2* markers. The panel for the collection included candidate 5A frost-tolerance markers, development-linked loci, and three independent non-*Fr* markers developed within the same breeding program (a summary of markers, allele classes, and sources is given in **Table 2**):

**Table 2:** Molecular markers used to assess frost tolerance in winter durum wheat: locus, assay type, allele classes, marker source, and observed direction of effect in the studied collection.

Marker	Locus/gene	Type	Allele classes (dye = base)	Source of marker (assay and physical position)	Direction observed in this collection
S2269949	*Fr-A2*, *CBF-A14* haplotype (5A)	KASP (SNP tagging the *CBF-A14* copy-number haplotype in the source germplasm)	FAM = A/HEX = G	Sieber et al., 2016; Würschum et al., 2017	Major class (FAM) higher survival, **Table 4**
S1077313	*Fr-A2*, *CBF-A14* haplotype (5A)	KASP (SNP tag)	FAM = G/HEX = A	Sieber et al., 2016; Würschum et al., 2017	Major class (HEX) higher survival, **Table 4**
S1862541	*Fr-A2*, *CBF-A14* haplotype (5A)	KASP (SNP tag)	FAM = G/HEX = C	Sieber et al., 2016; Würschum et al., 2017	Major class (HEX) higher survival, **Table 4**
S1298957	*Fr-A2*, *CBF-A14* haplotype (5A)	KASP (SNP tag)	FAM/HEX	Sieber et al., 2016; Würschum et al., 2017	Major class (HEX) higher survival, **Table 4**
S1051014	*Fr-A2*, non-CNV component (5A)	KASP (SNP)	FAM = C/HEX = G	Würschum et al., 2017	No difference (*p* = 0.442)
Vrn1_new	*Fr-A1*/*VRN-A1*, exon 4 (5A)	KASP (SNP)	*vrn-A1a* short/*vrn-A1b* long	Rasheed et al., 2016; nomenclature Giménez et al., 2025	*vrn-A1b* higher survival (*p* = 0.019)
VrnA1_9K0001	*VRN-A1*, growth habit (5A)	KASP (SNP)	Monomorphic (winter)	Rasheed et al., 2016	Not applicable
Vrn-B1	*VRN-B1* (5B)	PCR + electrophoresis	Monomorphic	Fu et al., 2005	Not applicable
Mor534 = 5B_517276534	Non-*Fr* locus (5B)	KASP (SNP)	FAM = A/HEX = G	Divashuk et al., 2026 (assay and position)	G higher survival (+8.2 percentage points, *p* = 0.052)
Mor587 = 1B_41099587	Non-*Fr* locus (1B)	KASP (SNP)	FAM = T/HEX = C	Divashuk et al., 2026 (assay and position)	T higher survival (+7.1 percentage points, *p* = 0.048)
Mor866 = 7B_598228866	Non-*Fr* locus (7B)	KASP (SNP)	FAM = C/HEX = A	Divashuk et al., 2026 (assay and position)	No difference (*p* = 0.860)

- Fr-A2, markers tagging the *CBF-A14* copy-number haplotype: S2269949, S1077313, S1862541, and S1298957 (Sieber et al., 2016; Würschum et al., 2017);
- Fr-A2, non-CNV component: S1051014 (Würschum et al., 2017);
- Fr-A1/*VRN-A1*: VrnA1_9K0001 (growth-habit diagnostics) and Vrn1_new (exon 4, short/long vernalization) (Rasheed et al., 2016; nomenclature per Giménez et al., 2025);
- VRN-B1 (5B) (Fu et al., 2005);
- Independent non-*Fr* loci: Mor534 = 5B_517276534 (5B), Mor587 = 1B_41099587 (1B), and Mor866 = 7B_598228866 (7B) (Divashuk et al., 2026). The allele-specific and common primers of these three assays are identical to those published in **Table 2** of Divashuk et al. (2026), and the allele-specific bases lie on the same strand as the reference and alternative alleles reported there, so the dye assignment is unambiguous: Mor587 FAM = T and HEX = C, Mor534 FAM = A and HEX = G, Mor866 FAM = C and HEX = A.

For KASP, a 10-µL reaction contained 5 µL of Master mix KBS-1050-102 (FAM, HEX, ROX dyes; LGC Biosearch Technologies, UK), 1.5 pM allele-specific primers, 3.75 pM common primer, and 5 µL of template DNA (50 ng per well). The amplification regime was as follows: 15 min at 94 °C; 10 cycles (20 s at 94 °C; 60 s at 61 °C, decreasing by 0.6 °C per cycle); 35 cycles (20 s at 94 °C; 60 s at 55 °C); signal read for 60 s at 37 °C. PCR was run on a Bio-Rad CFX96 instrument (Bio-Rad Laboratories, USA), and the data were analyzed in Bio-Rad CFX Manager 3.1. The resulting genotype calls for all 161 accessions of the studied collection across the 11 markers are given in **Supplementary Table S1**.

The *VRN-B1* marker was typed by PCR followed by electrophoresis in a 1.5% agarose gel (Tris-borate-EDTA buffer): the primer pair Intr/B/F + Intr1/B/R3 gives the *Vrn-B1* amplicon of 709 bp, and Intr/B/F + Intr1/B/R4 gives a *vrn-B1* of 1,149 bp (Fu et al., 2005). A 25-µL reaction contained 70 mM Tris-HCl (pH 9.3), 16.6 mM (NH_4_)_2_SO_4_, 2.5 mM MgCl_2_, 0.2 mM of each dNTP, 50 pM of each forward and reverse primer (Syntol LLC, Moscow), 0.05 U/µL Taq polymerase (Sileks, Moscow), and 100 ng of DNA; PCR amplification was performed using a Bio-Rad T100 thermocycler. Following electrophoresis, the gels were stained with ethidium bromide and visualized using a Gel Doc XR+ imaging system (Bio-Rad).

Assay quality control: Call rates per marker in the collection ranged from 73% to 100% (**Table 3**). Forty-one duplicate pairs across six assays, re-run on a second plate, were 100% concordant. In the F_2_, the call rate was 98.0% at both markers.

**Table 3:** Markers in the studied collection.

Marker	Locus/component	*n*	MAF	He	Classes (number of lines)	Status	Call rate, %
S2269949	*Fr-A2*, *CBF-A14* haplotype (5A)	160	0.056	0.106	FAM (A) 151/HEX (G) 9	Nearly fixed	99
S1077313	*Fr-A2*, *CBF-A14* haplotype (5A)	161	0.037	0.072	HEX (A) 155/FAM (G) 6	Nearly fixed	100
S1862541	*Fr-A2*, *CBF-A14* haplotype (5A)	159	0.050	0.096	HEX (C) 151/FAM (G) 8	Nearly fixed	99
S1298957	*Fr-A2*, *CBF-A14* haplotype (5A)	159	0.082	0.150	HEX 146/FAM 13	Nearly fixed	99
S1051014	*Fr-A2* non-CNV (5A)	118	0.483	0.499	FAM (C) 61/HEX (G) 57	Polymorphic	73
Vrn1_new	*Fr-A1*/*VRN-A1*, exon 4 (5A)	161	0.130	0.227	Long/*vrn-A1b* 140/short/*vrn-A1a* 21	Polymorphic	100
VrnA1_9K0001	*VRN-A1*, growth habit (5A)	161	0.000	0.000	Winter 161	Monomorphic	100
Vrn-B1	*VRN-B1* (5B)	161	0.000	0.000	Winter 161	Monomorphic	100
Mor534	Non-*Fr* (5B)	152	0.211	0.332	G 120/A 32	Polymorphic	94
Mor587	Non-*Fr* (1B)	156	0.308	0.426	T 108/C 48	Polymorphic	97
Mor866	Non-*Fr* (7B)	161	0.366	0.464	A 102/C 59	Polymorphic	100

### 2.7 Statistical analysis

The percentage of surviving plants was modeled with a linear mixed model after an angular (arcsine) transformation of the response, which stabilizes the variance of a proportion. The model was.

(1) $${\text{z}}_{\text{ij}}=\text{μ}+{\text{α}}_{\text{g}}\left(\text{i}\right)+{\text{γ}}_{\text{y}}\left(\text{ij}\right)+{\text{δ}}_{\text{r}}\left(\text{ij}\right)+{\text{b}}_{\text{i}}+{\text{ϵ}}_{\text{ij}}$$

where z_ij_ = arcsin(√(P_ij_/100)) is the angular-transformed percentage of survivors P_ij_ for the jth observation of accession *i*; μ is the overall intercept; α_g(i)_, γ_y(ij)_, and δ_r(ij)_ are the fixed categorical effects of the marker genotype *g*, the year *y*, and the freezing regime *r*, respectively; *b_i_* is the random intercept of accession *i*, *b_i_* ~ *N*(0, σ²*_b_*); and ϵ_ij_ is the residual error, ϵ_ij_ ~ N(0, σ²). Fixed effects and variance components were estimated by restricted maximum likelihood (REML).

Estimated marginal means (EMMs) per allele group were reported, back-transformed to percentages as *P* = 100 · sin²(*z*), together with pairwise contrasts and 95% confidence intervals (CIs). Marker × freezing-regime interaction terms were examined by likelihood-ratio tests against the additive model and showed no consistent dependence; thus, they were not included in the reported models. Year and freezing regime enter as additive main effects: the year × regime layout is incomplete (12 of the 20 possible combinations are represented, and 5 of them occur in a single year or under a single regime); hence, a saturated environment factor was not fitted. Interaction with the previous crop was not assessed, because the previous crop is constant in the dataset (fallow). Throughout, *n* is the number of observations (accession × year × regime) or the number of accessions, as stated.

We report *p*-values from the primary mixed model together with effect sizes, given as EMMs with 95% CIs and as the contrast between allele classes in percentage points with its 95% CI. The *p*-values of the primary model are the basis of our inference. The 11 markers were not drawn from a large set: each was selected *a priori* on published evidence linking it to frost tolerance and constitutes a separate hypothesis; thus, this is a targeted assessment of known candidate loci rather than an exploratory screen, and the per-comparison error rate is the quantity of interest. Because the five polymorphic markers were tested against the same trait in the same dataset, Holm- and Benjamini–Hochberg-adjusted *p*-values are reported alongside the unadjusted values at the request of the reviewers, so that a reader can apply a family-wise standard; they are not used as a criterion here. The effects at 1B, 5B, and VRN-A1 are described as nominal and the markers are described as candidates, because they were not tested in segregating material. The repeatability of the survival measurement was estimated from the variance components of the primary model. Because the archived data hold the percentage of survivors and not the underlying counts, a binomial model could not be fitted. The primary model was therefore refitted on the empirical-logit scale and on the untransformed percentage scale (**Supplementary Table S10**): the sign and size of every effect are unchanged on all three scales, and only for Mor587 does the *p*-value move across 0.05 (0.048, 0.081, and 0.028 on the angular, empirical-logit, and percentage scales), which is one more reason to treat 1B and 5B as candidates.

For every marker, the minor allele frequency (MAF) and expected heterozygosity (He = 2pq) were computed. The four *Fr-A2* markers (nearly fixed, minor class 6 to 13 lines) and the monomorphic markers (VrnA1_9K0001 and *VRN-B1*) were reported descriptively only. The polymorphic markers had two homozygous classes; hence, the additive, dominant, and recessive models coincide with the two-class contrast. Pairwise association between polymorphic loci was assessed with Cramér’s *V*; it indicates weak statistical association in the collection studied and does not, by itself, prove independent inheritance, recombination frequency, or physical linkage of the loci.

The association of frost tolerance with heading date was assessed with the same mixed model, entering the centered heading date (DOY) as a continuous accession-level covariate, as shown in **Equation 2**:

(2) $${\text{z}}_{\text{ij}}=\text{μ}+\text{β}\cdot {\text{DOY}}_{\text{i}}+{\text{γ}}_{\text{y}}\left(\text{ij}\right)+{\text{δ}}_{\text{r}}\left(\text{ij}\right)+{\text{b}}_{\text{i}}+{\text{ϵ}}_{\text{ij}}$$

where β is the regression coefficient on the centered heading date DOY*_i_* of accession *i*, and γ*_y_*_(_*_ij_*_)_, δ*_r_*_(_*_ij_*_)_, *b_i_*, and ϵ*_ij_* are as in Equation 1. Heading date enters at the accession level, whereas year and freezing regime describe the environment of the individual observation.

The inverse association of the regrowth score with heading date was assessed by Spearman’s rank correlation within each year.

Because individual plant identities could not be reliably matched between the genotyping before freezing and the later sampling of survivors, the association between *Fr-A2* alleles and survival in the F_2_ panel was assessed at the family level with a within-family randomization test. For each marker, the observed mean dose of the adapted-parent allele among the genotyped survivors was compared with a null distribution generated from 100,000 random draws. In each draw, the same number of plants as the observed survivors of that family was sampled without replacement from the pre-selection genotypes within each family, and the doses were pooled across families. The test uses family membership only: it makes no assumption of independence between plants, no asymptotic approximation, and no use of individual plant identity, and it is by construction a within-family test; hence, differences in allele frequency between families cannot contribute. Families monomorphic at a marker contribute a constant to every draw and do not affect the test; thus, all eight families enter both tests. The direction of the hypothesis, enrichment of the adapted-parent allele among survivors, was specified in advance; the one-sided *p* is the fraction of draws with a mean dose at least as high as observed, and the two-sided *p* is twice the smaller tail. A combined dose over the two markers was not used, because tightly linked markers belong to one locus and their summation would inflate significance.

Computations were performed in Python (3.11 to 3.12) with pandas (2.3 to 3.0) and numpy 2.4 for data processing and the calculation of allele doses, statsmodels (0.14 to 0.15) for the linear mixed models (statsmodels.formula.api.mixedlm, REML estimation) and the Holm and Benjamini–Hochberg corrections (statsmodels.stats.multitest), scipy 1.17 for likelihood-ratio tests, Spearman rank correlations and Fisher exact tests, and numpy for the within-family randomization test; figures were drawn with matplotlib (3.10 to 3.11). KASP calling was done in Bio-Rad CFX Manager 3.1. The datasets on which the analyses rest are included in the **Supplementary Material** (Data availability statement).

## 3 Results

### 3.1 Variation in frost tolerance

Frost tolerance depended strongly on the environment, that is, on the combination of year and freezing regime or series. Mean survival across environments ranged from approximately 4% (−16 °C, 2022) to approximately 73% (−15 °C, 2020); data by environment are given in **Table 1**. This wide spread dictated analysis with mixed models that account explicitly for the environment. Full per-accession data are given in **Supplementary Table S2**.

### 3.2 Marker polymorphism in the collection

Within the studied collection, the favorable *Fr-A2* variation is nearly fixed at all four markers tagging the *CBF-A14* copy-number haplotype. The MAF ranged from 0.04 to 0.08 across the four markers, and the minor class comprises 6 to 13 lines even at near-complete coverage (call rate 99%–100%). The non-CNV component of *Fr-A2* (S1051014), by contrast, is highly polymorphic (MAF 0.48, He 0.50). Growth habit is uniformly winter, as expected: the marker VrnA1_9K0001 is monomorphic (all 161 accessions winter), and *VRN-B1* is also monomorphic (**Table 3** and **Figure 1A**). The exon-4 marker Vrn1_new is moderately polymorphic (MAF 0.13); the long-vernalization allele predominates (*vrn-A1b*, “2147 type”; 140 lines), which means that the allele associated with higher frost tolerance predominates in the collection (Section 3.3). The short-vernalization allele is minor (*vrn-A1a*, “Jagger type”; 21 lines).

**Figure 1:**
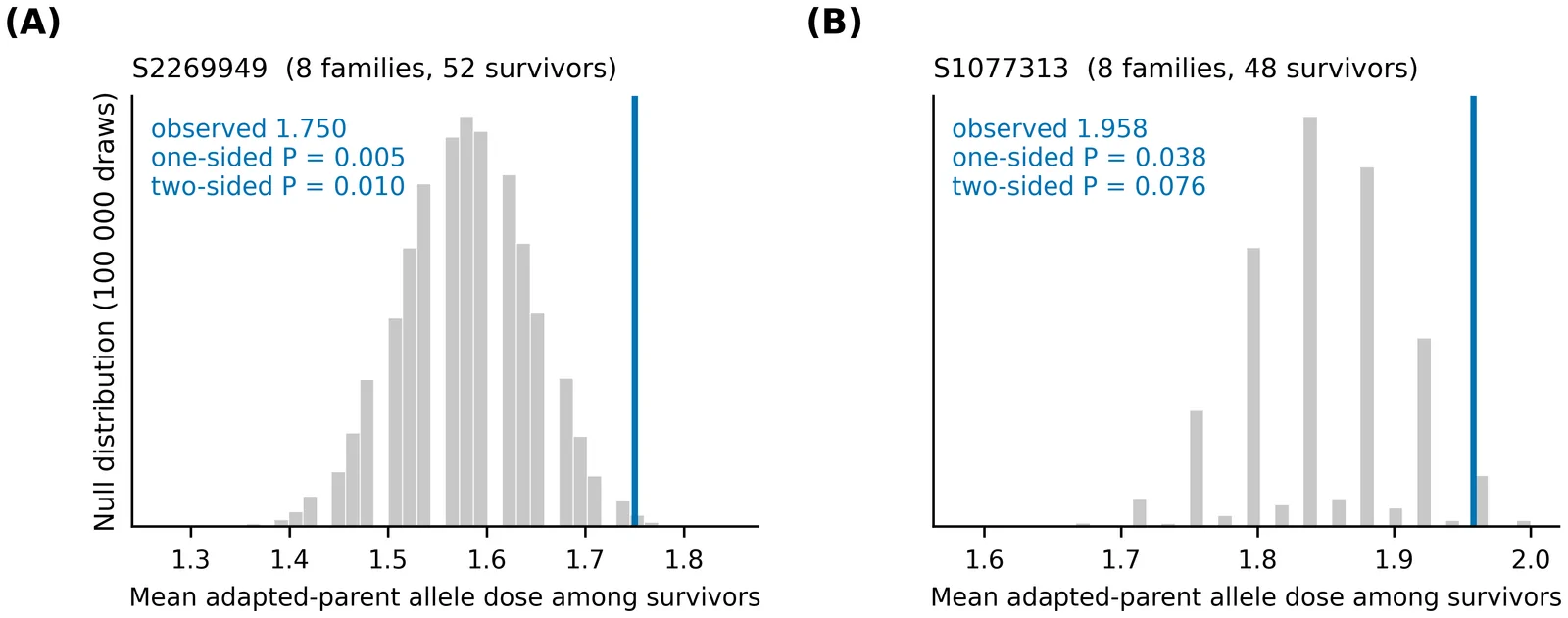
Dose of the adapted-parent *Fr-A2* allele among surviving F_2_ plants against the null distribution of the within-family randomization test (100,000 draws, stratified by family). **(A)** S2269949; **(B)** S1077313. The vertical line marks the observed mean dose among the survivors; the histogram is the null distribution obtained by drawing survivor-sized subsets within each family. One-sided *p* is the fraction of draws with a mean dose at least as high as observed; two-sided *p* is twice the smaller tail.

Unlike the nearly fixed *Fr-A2*, three independent markers (Divashuk et al., 2026) are markedly polymorphic in this near-complete sampling of the cultivar pool. Their minor allele frequencies were 0.21 for Mor534 (5B; He 0.33), 0.31 for Mor587 (1B; He 0.43), and 0.37 for Mor866 (7B; He 0.46) (**Table 3**; **Figure 1A**). Genetic variation at these loci in the adapted elite pool is therefore not exhausted. Pairwise Cramér’s *V* between these loci, and with the *Fr-A2*/*VRN-A1* markers, did not exceed 0.32 (the largest value, 0.317, was between S1051014 and Mor534, *n* = 111). Values of this order indicate no appreciable linkage disequilibrium in the sample studied, consistent with the markers residing on different chromosomes (1B, 5B, and 7B); the value itself does not characterize recombination frequency.

### 3.3 Association of markers with frost tolerance

The four nearly fixed *Fr-A2* markers were assessed descriptively, because the minor class comprises only a few lines. At every marker, the minor class survived far less well than the major class: 7.3% against 44.3% at S2269949, 8.9% against 43.7% at S1077313, 8.2% against 44.4% at S1862541, and 13.5% against 45.4% at S1298957 (**Table 4**). The likelihood-ratio test for the genotype term gives *p* from 1.2 × 10^−12^ to 8.8 × 10^−7^. This comparison is confounded with origin and is not an unbiased estimate of the locus effect: of the 15 lines that carry a minor class at one or more of the four markers, 7 are foreign, 3 come from other Russian programs, 3 come from the Donskoy program, and only 2 come from the Krasnodar program that contributes 101 of the 161 accessions. What it does establish within this collection, independently of the source publications, is the direction. The class that is fixed in the Russian pool is the class associated with higher survival. The four markers are largely concordant with one another, with pairwise concordance from 95.6% to 98.1%, and four lines carry the minor class at all four (**Supplementary Tables S5a**, **b**). The polymorphic markers behaved as follows (**Table 5**; **Figure 1B**).

**Table 4:** Survival of the minor class at the nearly fixed *Fr-A2* markers. *p* is the likelihood-ratio test for the genotype term in the primary model.

Marker	Allele class	Lines	Mean survival, %	SD	Model EMM, %	*p*
S2269949	Major (FAM, A)	151	44.3	16.2	43.9	
	Minor (HEX, G)	9	7.3	10.6	5.1	5.9 × 10^−12^
S1077313	Major (HEX, A)	155	43.7	17.0	43.0	
	Minor (FAM, G)	6	8.9	12.8	7.2	8.8 × 10^−7^
S1862541	Major (HEX, C)	151	44.4	16.3	43.7	
	Minor (FAM, G)	8	8.2	10.9	6.0	4.9 × 10^−10^
S1298957	Major (HEX)	146	45.4	15.3	44.9	
	Minor (FAM)	13	13.5	18.7	9.0	1.2 × 10^−12^

SD, standard deviation; EMM, estimated marginal mean.

**Table 5:** Estimated marginal mean survival and marker associations with frost tolerance in the winter durum wheat collection.

Marker	Locus	*n*	EMM % by class	Difference, percentage points [95% CI]	*p*	*p* (Holm)	*q* (BH)
S1051014	*Fr-A2* non-CNV	118	FAM (C) 38.9/HEX (G) 41.9	+3.0 [−4.7, +10.9]	0.442	0.883	0.552
Vrn1_new	*Fr-A1*/*VRN-A1*, exon 4	161	Long 43.1/short 31.7	+11.4 [+1.9, +20.2]	0.019	0.096	0.087
Mor534 (5B)	Non-*Fr*	152	A 35.0/G 43.1	+8.2 [−0.1, +16.6]	0.052	0.194	0.087
Mor587 (1B)	Non-*Fr*	156	C 36.7/T 43.8	+7.1 [+0.1, +14.4]	0.048	0.194	0.087
Mor866 (7B)	Non-*Fr*	161	A 41.4/C 42.0	+0.6 [−6.1, +7.5]	0.860	0.883	0.860

BH, Benjamini–Hochberg.

The non-CNV component S1051014, despite its high polymorphism, showed no association with frost tolerance. EMMs were 38.9% (FAM class) versus 41.9% (HEX class), *p* = 0.442 (*n* = 118).

The exon-4 marker Vrn1_new showed an effect. Lines with the long-vernalization allele (*vrn-A1b*, “2147 type”) survived better: EMM 43.1% against 31.7% for lines with the short-vernalization allele (*vrn-A1a*, “Jagger type”). The difference was 11.4 percentage points (95% CI + 1.9 to +20.2), *p* = 0.019, *n* = 161. The Holm-adjusted value is *p* = 0.096 and the Benjamini–Hochberg *q* = 0.087 (**Table 5**).

Two of the three independent non-*Fr* loci showed a nominal effect. At Mor587 (1B), allele T gave EMM 43.8% against 36.7% for allele C (difference +7.1 percentage points, 95% CI + 0.1 to +14.4, *p* = 0.048, *n* = 156). At Mor534 (5B), allele G gave EMM 43.1% against 35.0% for allele A (difference +8.2 percentage points, 95% CI −0.1 to +16.6, *p* = 0.052, *n* = 152). Mor866 (7B) showed no association with frost tolerance (EMM 41.4% for A against 42.0% for C, *p* = 0.860, *n* = 161). The Holm- and Benjamini–Hochberg-adjusted values are given in **Table 5** for reference (*p* = 0.194 by Holm and *q* = 0.087 for both loci); we describe 1B and 5B as candidate markers because their effects are nominal and were not tested in segregating material. The direction of the effect in this collection is the opposite of the one reported in the study that developed these assays. In the present collection, the higher-survival class is T at Mor587 (1B) and G at Mor534 (5B). In the source study (Divashuk et al., 2026), the favorable alleles at these loci are reported as C at 1B_41099587 and A at 5B_517276534, that is, the opposite class at both. Both markers lie in intergenic regions and act as tag markers, and the linkage phase between a tag marker and the causal variant can differ between populations (Section 4). Adjustment for the originating program does not remove the two signals. With a four-level origin group in the model, the values are *p* = 0.007 at Mor587 and *p* = 0.017 at Mor534. Within the Krasnodar program alone, they are *p* = 0.162 and *p* = 0.279 (**Supplementary Table S8**).

### 3.4 Segregation of Fr-A2 in F2

The F_2_ populations then present in the breeding process at the P.P. Lukyanenko National Grain Center, from crosses of adapted cultivars with foreign lines contrasting at the *Fr-A2* markers, were used to test the markers against survival, because in the collection, the favorable *Fr-A2* allele is fixed and the allele–trait association cannot be tested there (Section 3.2).

The F_2_ panel comprised 152 plants from eight families genotyped before freezing, of which 52 survived and were genotyped again (survival 33.6%, range 5.9 to 70.0% by family, **Supplementary Table S6**). Seven of the eight families are three-way crosses (Section 2.1); hence, the expected dose of the adapted-parent allele before selection is 1.50 in those families rather than 1.00; the observed doses before freezing, 1.510 at S2269949 and 1.752 at S1077313, are consistent with this structure. The dose of the adapted-parent allele rose at S2269949 from 1.510 before freezing to 1.750 among survivors (within-family randomization test, one-sided *p* = 0.005, two-sided *p* = 0.010; 8 families, 149 plants called before freezing, and 52 survivors). At S1077313, the dose rose from 1.752 to 1.958 (one-sided *p* = 0.038, two-sided *p* = 0.076; 8 families, 149 plants, and 48 survivors). The direction of the hypothesis, enrichment of the adapted-parent allele among survivors, was specified before the test; the two-sided values are given for a reader who prefers not to use it. S1077313 called 1 heterozygote among 149 plants, against 41.5 expected from the pedigrees, and three families (3321-3, 3321-5, and 3321-7) carry a single class at this marker; these families contribute a constant to every draw and do not affect the test, but the marker is the weaker of the two estimates of the locus. S2269949 segregates in line with the pedigrees: against the pedigree-derived ratio, five of the eight families give *p* > 0.10, two give *p* between 0.05 and 0.10 (0.056 and 0.096), and one gives *p* = 0.038 (**Supplementary Table S7**). The two markers are tightly linked and tag the same *Fr-A2* block; thus, these are two estimates of one locus. The pre-selection and the survivor blocks were genotyped in different years and on different plates, and the rate of heterozygous calls does not differ between them (23.5% against 17.3% at S2269949, Fisher *p* = 0.44); hence, the shift is not a batch artifact. The same *Fr-A2* markers that are nearly fixed in the full collection retain an association with survival under freezing in segregating material (**Table 6**; **Figure 2**).

**Table 6:** F_2_ panel: dose of the adapted-parent *Fr-A2* allele before freezing and among survivors, with the within-family randomization test (100,000 draws; one-sided *p* for the pre-specified direction, two-sided *p* as twice the smaller tail).

Marker	Adapted-parent allele	Families	Plants before freezing	Survivors	Mean dose (before → survivors)	*p* (one-sided)	*p* (two-sided)
S2269949	FAM	8	149	52	1.510 → 1.750	0.005	0.010
S1077313	HEX	8	149	48	1.752 → 1.958	0.038	0.076

**Figure 2:**
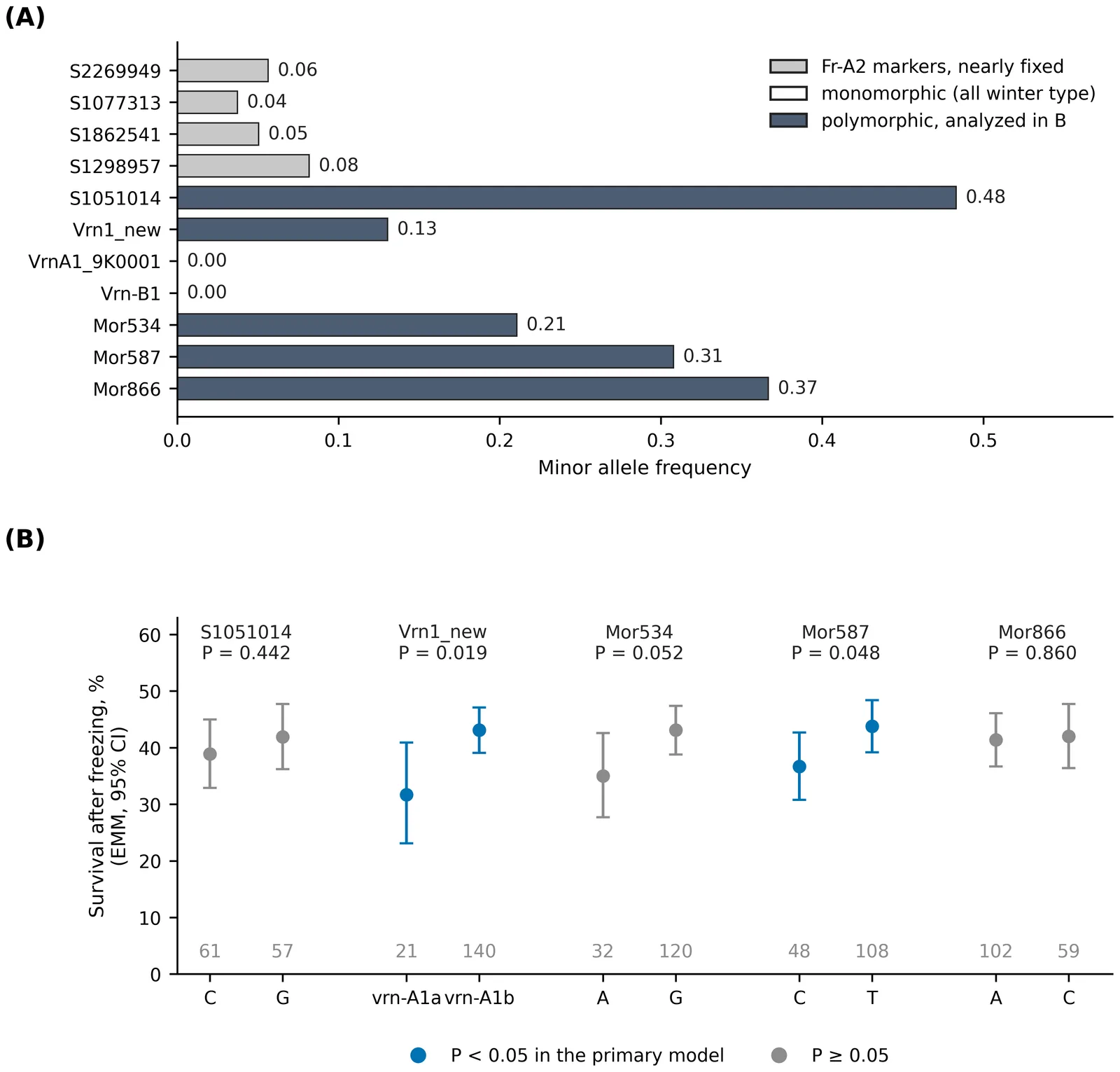
Summary of markers in the studied collection (*n* = 161). **(A)** Polymorphism: minor allele frequency across all 11 markers, contrasting the nearly fixed *Fr-A2* markers and the monomorphic loci with the segregating independent non-*Fr* loci 1B/5B/7B and the non-CNV component S1051014 (descriptive contrast, direct count from the collection data). **(B)** Survival by allele class for the five polymorphic markers: EMMs (%) from the primary mixed model with 95% CIs (Section 2.7). The *p*-value above each pair is that of the primary model; pairs with *p* < 0.05 in the primary model are drawn in blue, the others in gray; the number of lines in each class is given at the foot of the panel. Nearly fixed and monomorphic markers are not shown in panel B because there is no variance to estimate an effect.

### 3.5 Frost tolerance and developmental timing

Within the studied collection, frost tolerance was associated with heading date (per-accession mean heading dates from the Russian nursery in **Supplementary Table S11**). Later heading was accompanied by higher survival, with a slope on the angular scale of +0.0142 per day (95% CI + 0.0064 to +0.0220, *p* = 0.0004, *n* = 155 accessions, **Figure 3**). Heading dates in the sample spanned approximately 20 days (DOY 126 to 146, interquartile range approximately 7 days). At the mean survival level of the collection (45%), a shift of 10 days corresponds to about +14.1 percentage points of survival (+6.4 to +21.7 by the confidence limits of the slope), that is, about +1.4 percentage points per day. The polymorphism of vernalization length does not explain this association. Adding the Vrn1_new class to the model leaves the slope at +0.0135 per day (*p* = 0.0006), and the two exon-4 classes do not differ in heading date (134.2 against 133.0 DOY, Mann–Whitney *p* = 0.39). The association is carried largely by the contrast between breeding programs. With the origin group in the model, the slope falls to +0.0039 per day (*p* = 0.37), and within the Krasnodar program alone, it is +0.0060 per day (*p* = 0.13, *n* = 97 accessions). We therefore do not treat the slope as a quantitative guide for selection. No *Ppd-A1* or *Ppd-B1* marker was included in the panel; therefore, a photoperiod contribution was not tested, and a systematic difference in developmental stage at freezing between early- and late-heading accessions, which were sown on one date and frozen at tillering, cannot be excluded either. The regrowth score after freezing is associated with late heading in the opposite direction, with a negative Spearman rank correlation within each year.

**Figure 3:**
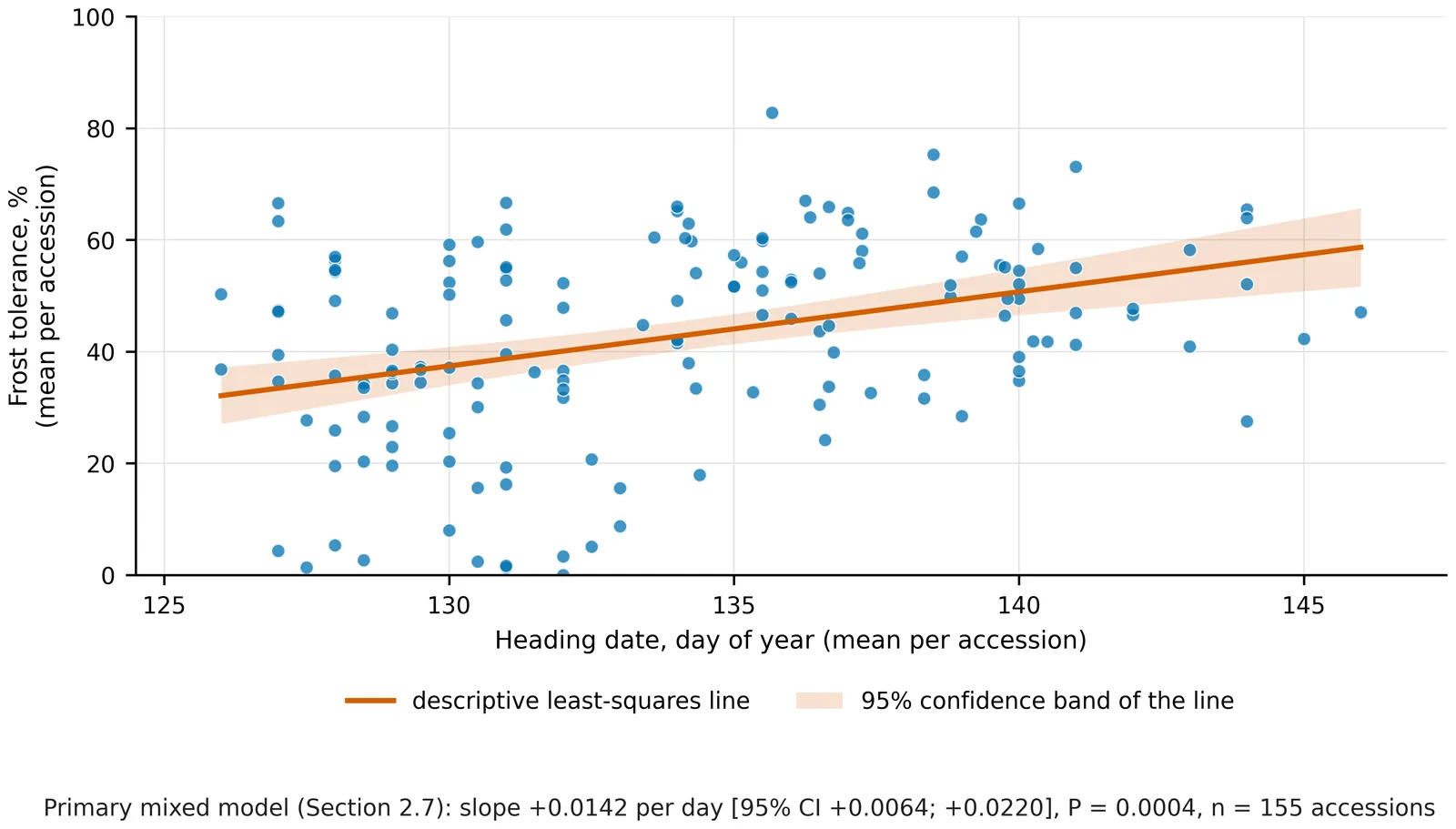
Frost tolerance as a function of heading date [day of year (DOY)] in the studied collection (*n* = 155; heading dates from the Russian nursery, **Supplementary Table S11**). Points are the mean per-accession frost tolerance (direct count); the line is the descriptive ordinary least squares (OLS) fit with its 95% confidence band; the primary mixed-model estimate is given below the plot: slope +0.0142 per day on the angular scale (95% CI + 0.0064 to +0.0220), *p* = 0.0004.

## 4 Discussion

The collection studied is a single, near-complete cultivar pool of Russian winter durum wheat that has undergone decades of selection for overwintering in the Krasnodar school of breeding for winter hardiness (the Lukyanenko school; Puchkov and Nabokov, 2001). In this pool, the favorable *Fr-A2* variation is nearly fixed. The *CBF-A14* copy-number component is close to fixation (He 0.07–0.15, minor class 6 to 13 lines even at near-complete coverage), while the non-CNV component S1051014, although highly polymorphic (He ≈ 0.50), is not associated with frost tolerance (*p* = 0.442). Selection for frost tolerance has always operated in this program; the favorable *Fr-A2* allele became fixed, and the segregating variation was exhausted. The favorable *Fr-A2* class is present across the whole breeding period: its frequency rises from 0.60 to 0.87 among accessions entered into State Variety Trials before 2000 to 0.95 to 1.00 among those entered after 2010, at all four markers, while none of the five control loci shows a trend (**Supplementary Table S12** and **Supplementary Figure S6**). The favorable class was thus already predominant in the oldest accessions and approached fixation over the breeding period, which argues against an explanation by a recent bottleneck. Within the collection, there is almost no variation at these markers; thus, their effect cannot be assessed here; the markers themselves nevertheless work, as shown below.

The same locus behaves differently in heterogeneous material. In a pan-European analysis of durum wheat (Habib et al., 2026), frost tolerance is almost entirely between-group. East European accessions survived on average at 61.1%, South European ones at only 7.33%, and chromosome 5A proved to be the main zone of between-group divergence (34 of 62 F_ST_ outliers across the whole panel). When a locus is itself one of the main factors of population structure, correcting for structure in a genome-wide analysis cannot fully separate its own contribution, and the estimated 5A contribution is reasonably read as an upper bound. In Habib et al. (2026), *Fr-A2* is confounded with population structure, whereas in our adapted collection, it is fixed.

The informativeness of a marker depends not only on the biology of the locus but also on the gene pool in which it is assessed. In composite collections of accessions of different origin, the locus effect is statistically confounded with population structure. A marker aligned with the main axis of structure will show a strong association with the trait even where the locus’ own contribution is small. In segregating material (F_2_), the same locus is tested fairly, because meiosis reshuffles genetic backgrounds, and enrichment of the favorable allele among survivors points directly to its action. In an adapted elite gene pool that has undergone long selection for overwintering, useful variation may prove exhausted and fully fixed. The absence of a signal then says only that little variation remains in the pool (Maccaferri et al., 2019; Royo et al., 2020). We therefore turned to segregating material to establish that a working locus, and not chance, lies behind the fixation. In our F_2_ panel from crosses of adapted and foreign lines, the dose of the adapted-parent *Fr-A2* allele among plants that survived freezing rose from 1.510 to 1.750 at S2269949 (one-sided *p* = 0.005, two-sided *p* = 0.010) and from 1.752 to 1.958 at S1077313 (*p* = 0.038 and 0.076). The same conclusion is independently supported by the backcross test of Habib et al. (2026): in their BC_2_F_2_ populations, the *Fr-A2* marker S1862541, which tags the same locus, was tightly associated with survival (χ² = 54.3; *p* ≈ 1.6 × 10^−12^; Kruskal–Wallis test, df = 2; the “KASP validation” section and **Supplementary Table S5** in Habib et al., 2026). Our F_2_ was genotyped with S2269949 and S1077313, not with S1862541; hence, the agreement is at the level of the locus, tested with different markers of the same *CBF-A14* haplotype, and not at the level of a shared marker. The agreement of two independent segregating materials at one locus indicates that *Fr-A2* is real and that its fixation in the collection is the result of directional selection rather than a founder effect. Drift or a founder effect would fix an arbitrary allele; here, the favorable allele is fixed, and its frequency rose over the breeding period at all four markers and at none of the five control loci (**Supplementary Table S12**), in a program with documented long-term selection for overwintering. Drift from an already high founding frequency cannot be excluded at a single locus, but it does not predict a concordant rise at four markers of one haplotype with no trend at the control loci.

Our *Fr-A2* markers tag the canonical *CBF* cluster of *Fr-A2* (≈516–523 Mb), whereas the genome-wide 5A peak *QFt-5A* in Habib et al. (2026) (≈488 Mb) and the nearby 5A peak of Divashuk et al. (2026) lie approximately 30 Mb more proximal. Whether this is a single extended linkage block or two distinct determinants remains open and requires fine mapping; our conclusion about near-fixation applies to the *CBF*-tagged part of the locus. The relation between these SNP assays and *CBF-A14* copy number was established in the source germplasm (Sieber et al., 2016; Würschum et al., 2017) and has not been verified by copy-number measurement in Russian winter durum, which is a limitation of the present study.

Beyond *Fr-A2*, the picture differs. The three independent non-*Fr* loci (1B, 5B, and 7B) are markedly polymorphic in the adapted pool (MAF 0.21–0.37); hence, variation outside *Fr* is not exhausted here. They lie on different chromosomes and therefore segregate independently, which makes pyramiding of favorable alleles possible; the statistical association (Cramér’s *V* ≤ 0.32) is an additional observation of the same order. Of these, 7B (Mor866) showed no association with frost tolerance. We read the discrepancy with Divashuk et al. (2026), where 7B was informative in segregating material, cautiously, as a set of possible explanations rather than an established cause. The contribution of a single locus depends on the genetic background and may not carry over from segregating material to a uniform adapted pool where the background has been leveled by long selection. Loci 1B (Mor587) and 5B (Mor534) gave a nominal effect of the same direction and similar magnitude: the favorable alleles (T at 1B and G at 5B) raised survival by approximately 7 to 8 percentage points (*p* = 0.048 and 0.052). This is relative tolerance, apparent already against the fixed *Fr-A2* background; in a pool where the *Fr-A2* contribution is uniform, differences at 1B and 5B become visible. The direction of the 1B and 5B effects in our collection is opposite to that reported in the companion study (Divashuk et al., 2026), where the favorable alleles are C and A, respectively. Both markers lie in intergenic regions and act as tag markers; thus, their linkage phase with the causal variant may differ between a segregating population from specific crosses and a heterogeneous adapted pool; a sign change of a tag-marker effect between populations is a known limitation of marker-assisted selection, and the direction requires confirmation in independent samples. This does not render the markers useless: within any given population the favorable allele simply needs to be re-established before selection begins. In the source study, the three markers were placed in intergenic regions of the reference genome: 1B_41099587 (approximately 41.1 Mb) is in tight linkage with a cluster of genes encoding histidine-containing phosphotransfer (HPt) proteins, intermediaries of two-component signaling that in *Arabidopsis* mediate cold and osmotic stress responses; 5B_517276534 (approximately 517.3 Mb) lies close to genes encoding ornithine decarboxylase (ODC1B) and phospholipase D delta (PLDδ), enzymes of polyamine synthesis and membrane remodeling under low temperature, approximately 25 to 30 Mb distal to the *Fr-B2* cluster; and 7B_598228866 (approximately 598.2 Mb) is adjacent to genes encoding the 40S ribosomal protein S14 (Divashuk et al., 2026). These are positional candidates inferred from proximity in the reference genome, not functionally validated genes.

The vernalization-length marker Vrn1_new gave an additional effect: lines with the long-vernalization allele (*vrn-A1b*) survived better (43.1% versus 31.7%, *p* = 0.019). We treat 1B and 5B, and to a lesser extent *VRN-A1*, as candidate markers that showed an effect and are potentially useful in breeding; their effects are nominal, they were not tested in segregating material in this study, and they require confirmation in independent samples. The molecular context is consistent with the polygenic, *CBF*-mediated nature of the trait: in winter cultivars, the expression of *CBF12* and *CBF14* rises under cold stress (Zainchkovskaya et al., 2025). Cold-tolerance determinants are described outside the fifth homoeologous group as well (Lei et al., 2023).

The practical selection strategy depends directly on the crossing plan. When external cultivars and lines differing at *Fr-A2* are introduced (introgression from contrasting, including non-adapted, material), the main reliance is on *Fr-A2*. There, the locus segregates and the *Fr-A2* markers work, as shown by two independent segregating materials at the same locus, our F_2_ and the BC_2_F_2_ of Habib et al. (2026). Within the already tolerant elite pool, where *Fr-A2* is fixed, the remaining lead is the candidate markers 1B and 5B (and possibly the long-vernalization *VRN-A1* allele); their effects are nominal and they were not tested in segregating material here; hence, their use for ranking lines by relative tolerance awaits that test. Joint accumulation of favorable non-*Fr* alleles (pyramiding) would be justified by their additive action. The replication cost of phenotyping is itself an argument for markers: the repeatability of a single freezing test is *R* = 0.45, and about five environments per accession are needed to reach *R* = 0.8 (Section 2.3), whereas a genotype is scored once. In segregating material (Divashuk et al., 2026; *n* = 270), the probability of survival rose almost linearly with the number of favorable 1B/5B/7B alleles (combined model, area under the receiver operating characteristic curve, AUC = 0.808), with no sign of epistasis. Screening with these markers is worthwhile in generations where homozygosity is sufficient for a reliable genotype call. Foreign introgressions, including rye translocations, may serve as an additional source of variation for durum wheat, although their use is constrained by effects on grain technological quality (Korobkova et al., 2023).

Frost tolerance in our material is also linked to developmental timing, and this link is a biological trade-off. Later heading was accompanied by higher survival (+0.0142 per day on the angular scale, *p* = 0.0004), whereas the regrowth score after freezing fell with later heading. Physiologically, this agrees with the classical two-phase theory of hardening (Maksimov, 1913; Trunova, 2007; Tumanov, 1979). A more “winter”, later-maturing phenotype holds the hardened state longer and therefore survives better, but comes with weaker spring regrowth. The same opposition between tolerance and rapid resumption of growth appears in observations linking frost tolerance to respiration and water-soluble carbohydrate content over the autumn–spring period (Pomortsev et al., 2013) and in indirect indicators of tolerance: enzymatic diagnostics, which use the activity of stress-related enzymes in hardened tissue (Statsenko and Blinokhvatov, 2019), and the cell-membrane permeability index of seedlings, an electrolyte-leakage measure (Gurova et al., 2021). These indicators track the physiological state of the hardened plant and correlate with survival, but they are measured on the plants whose survival is in question; thus, they diagnose rather than predict, which is the gap a DNA marker fills. Later heading also means later maturity. In the growing zone of winter durum wheat in southern Russia, late grain filling may coincide with summer heat and drought; hence, long-vegetation accessions risk yield losses, and the relative weight of overwintering and developmental speed differs among agroclimatic zones (cf. Royo et al., 2020). Therefore, despite the substantial magnitude of the association, heading date on its own cannot serve as a surrogate criterion for selection for frost tolerance: selecting only for lateness will improve survival but worsen regrowth. Frost tolerance is tuned within a complex of traits rather than at a single locus. Grain quality belongs to the same complex: in durum, it is a demanding trait system with its own loci and its own sensitivity to the environment, and it is screened separately in breeding programs (Díaz-Ceniceros et al., 2026; Shakir et al., 2026; Utebayev et al., 2026).

Limitations: The associations reported for the 1B and 5B markers are not corrected for relatedness. With 11 markers, no marker-based kinship correction is possible, and complete pedigrees are not available for the introduced material; thus, the present analysis is not better protected against population structure than the pan-European analyses it discusses. Box and tier identifiers were not archived and could not enter the model. The denominator of the survival percentage is not part of the archived dataset; hence, a binomial model could not be fitted; the sensitivity of the results to the analysis scale is reported in **Supplementary Table S10**. The heading-date analysis uses the records of the Russian nursery only (**Supplementary Table S11**). Individual plant identity was not carried through the F_2_ freezing test, and the F_2_ analysis therefore conditions on family membership alone. The F_2_ panel is small (152 plants, 52 survivors, and 8 families); therefore, the *Fr-A2* result is a single test of one locus and not an estimate of its effect size; a larger validation would require a new segregating population and a new freezing season. The 1B and 5B markers were not tested in segregating material, and the adjusted *p*-values in **Table 5** show that a reader applying a family-wise criterion would not call any of the collection associations significant.

## 5 Conclusions

In the cultivar pool of Russian winter durum wheat (East European breeding), the favorable *Fr-A2* class is nearly fixed at all four markers of the *CBF-A14* haplotype, and it has been present at high frequency across the whole breeding period. In segregating material, the dose of the adapted-parent *Fr-A2* allele among surviving plants rose at both markers, significantly at S2269949, which supports the reading that the fixation reflects selection on a working locus. The independent loci 1B (allele T) and 5B (allele G) are candidate markers with nominal effects of approximately 7 to 8 percentage points of survival against the fixed *Fr-A2* background; they were not tested in segregating material, and 7B is uninformative in this pool. Frost tolerance is linked to developmental timing, with later heading accompanying higher survival but weaker regrowth; thus, heading date cannot serve as a surrogate criterion. In practice, the choice of markers follows from the crossing plan: if *Fr-A2* is polymorphic in the breeding material, selection should rest on this locus; if *Fr-A2* is fixed, the candidate markers 1B and 5B and the long-vernalization *VRN-A1* allele are the leads to be validated in segregating material before use.

## Data Availability

The original contributions presented in the study are included in the article/**Supplementary Material**, further inquiries can be directed to the corresponding author/s.
