# Supplementary material for: The practical value of frost-tolerance markers in winter durum wheat breeding
Source: Front Plant Sci. 2026 Oct 21;17:1959465. doi: 10.3389/fpls.2026.1959465 (PMC13731339; doi:10.3389/fpls.2026.1959465)
Supplement: Supplementary file 1 [file DataSheet1.docx]

# Supplementary Material

The datasets underlying the analyses were added to this Supplementary Material at the reviewers' request (Tables S1 to S4 and S11); further data are available from the corresponding author on reasonable request.

Table S1. Genotypes of the studied collection: 161 accessions × 11 markers.

Correspondence of allele classes: the four *Fr-A2* markers (S2269949, S1077313, S1862541, S1298957) are KASP SNP assays tagging the *CBF-A14* copy-number haplotype in the germplasm of the source studies (Sieber et al., 2016; Würschum et al., 2017); their FAM/HEX classes are reported as called, and copy number was not measured in this study. For the SNP marker S1051014 the classes correspond to the nucleotide: FAM = C, HEX = G. Cells marked heterogeneous are accessions whose assay gave a mixed signal; they are excluded from every association model, as are heterozygous and failed calls.

| **sample_id** | **Cultivar / line** | **S2269949** | **S1077313** | **S1862541** | **S1298957** | **S1051014** | **Vrn1_new*** | **VrnA1_9K0001** | **Vrn-B1** | **Mor534** | **Mor587** | **Mor866** |
| --- | --- | --- | --- | --- | --- | --- | --- | --- | --- | --- | --- | --- |
| 20 | Krupinka | FAM (A) | HEX (A) | HEX (C) | HEX | HEX (G) | *vrn-A1b* | vrn-A1 Winter | *vrn-B1* (winter) | G | C | C |
| 40 | Krucha | FAM (A) | HEX (A) | HEX (C) | HEX | HEX (G) | *vrn-A1b* | vrn-A1 Winter | *vrn-B1* (winter) | G | heterogeneous | A |
| 203 | Kristall 2 | FAM (A) | HEX (A) | FAM (G) | heterogeneous | heterogeneous | *vrn-A1b* | vrn-A1 Winter | *vrn-B1* (winter) | A | C | A |
| 204 | Leukurum 21 | FAM (A) | HEX (A) | HEX (C) | HEX | HEX (G) | *vrn-A1b* | vrn-A1 Winter | *vrn-B1* (winter) | G | T | A |
| 205 | Alena | FAM (A) | HEX (A) | HEX (C) | HEX | HEX (G) | *vrn-A1b* | vrn-A1 Winter | *vrn-B1* (winter) | G | T | A |
| 206 | Kermen | FAM (A) | HEX (A) | HEX (C) | HEX | heterogeneous | *vrn-A1b* | vrn-A1 Winter | *vrn-B1* (winter) | A | T | A |
| 207 | Uniya | FAM (A) | HEX (A) | HEX (C) | HEX | HEX (G) | *vrn-A1b* | vrn-A1 Winter | *vrn-B1* (winter) | G | C | A |
| 208 | Zolotko | FAM (A) | HEX (A) | HEX (C) | HEX | HEX (G) | *vrn-A1b* | vrn-A1 Winter | *vrn-B1* (winter) | G | T | A |
| 209 | Laska | FAM (A) | HEX (A) | HEX (C) | HEX | FAM (C) | *vrn-A1b* | vrn-A1 Winter | *vrn-B1* (winter) | A | C | A |
| 210 | Odari | FAM (A) | HEX (A) | HEX (C) | HEX | heterogeneous | *vrn-A1b* | vrn-A1 Winter | *vrn-B1* (winter) | G | T | A |
| 211 | Altana | FAM (A) | HEX (A) | HEX (C) | HEX | HEX (G) | *vrn-A1b* | vrn-A1 Winter | *vrn-B1* (winter) | G | T | C |
| 212 | Kordon | FAM (A) | HEX (A) | HEX (C) | HEX | heterogeneous | Vrn-A1a | vrn-A1 Winter | *vrn-B1* (winter) | heterogeneous | heterogeneous | A |
| 213 | Dobryana | FAM (A) | HEX (A) | HEX (C) | HEX | FAM (C) | *vrn-A1b* | vrn-A1 Winter | *vrn-B1* (winter) | heterogeneous | T | A |
| 214 | Sinora | FAM (A) | HEX (A) | HEX (C) | HEX | FAM (C) | *vrn-A1b* | vrn-A1 Winter | *vrn-B1* (winter) | G | T | C |
| 215 | Donchanka | FAM (A) | HEX (A) | HEX (C) | FAM | heterogeneous | *vrn-A1b* | vrn-A1 Winter | *vrn-B1* (winter) | G | T | A |
| 216 | Kurant | FAM (A) | HEX (A) | HEX (C) | HEX | heterogeneous | *vrn-A1b* | vrn-A1 Winter | *vrn-B1* (winter) | A | T | C |
| 217 | Kristella | FAM (A) | HEX (A) | FAM (G) | HEX | FAM (C) | *vrn-A1b* | vrn-A1 Winter | *vrn-B1* (winter) | A | heterogeneous | A |
| 218 | Amazonka | FAM (A) | HEX (A) | HEX (C) | HEX | FAM (C) | Vrn-A1a | vrn-A1 Winter | *vrn-B1* (winter) | heterogeneous | T | C |
| 219 | Agat donskoy | FAM (A) | HEX (A) | HEX (C) | HEX | FAM (C) | *vrn-A1b* | vrn-A1 Winter | *vrn-B1* (winter) | G | T | A |
| 220 | Lazurit | FAM (A) | HEX (A) | HEX (C) | HEX | heterogeneous | *vrn-A1b* | vrn-A1 Winter | *vrn-B1* (winter) | G | T | A |
| 221 | Oniks | FAM (A) | HEX (A) | HEX (C) | HEX | HEX (G) | *vrn-A1b* | vrn-A1 Winter | *vrn-B1* (winter) | G | T | A |
| 222 | Diona | FAM (A) | HEX (A) | HEX (C) | HEX | heterogeneous | *vrn-A1b* | vrn-A1 Winter | *vrn-B1* (winter) | A | T | A |
| 223 | Kiprida | FAM (A) | HEX (A) | HEX (C) | HEX | heterogeneous | *vrn-A1b* | vrn-A1 Winter | *vrn-B1* (winter) | A | T | A |
| 224 | Teyya | FAM (A) | HEX (A) | HEX (C) | HEX | HEX (G) | *vrn-A1b* | vrn-A1 Winter | *vrn-B1* (winter) | G | T | A |
| 225 | Eyrena | FAM (A) | HEX (A) | heterogeneous | FAM | heterogeneous | *vrn-A1b* | vrn-A1 Winter | *vrn-B1* (winter) | G | T | A |
| 226 | Yakhont (760/10) | FAM (A) | HEX (A) | HEX (C) | HEX | heterogeneous | Vrn-A1a | vrn-A1 Winter | *vrn-B1* (winter) | G | C | C |
| 227 | Yubilyarka (330/10) | heterogeneous | HEX (A) | HEX (C) | HEX | FAM (C) | *vrn-A1b* | vrn-A1 Winter | *vrn-B1* (winter) | G | T | A |
| 228 | Yantarina | FAM (A) | HEX (A) | HEX (C) | HEX | heterogeneous | *vrn-A1b* | vrn-A1 Winter | *vrn-B1* (winter) | G | T | A |
| 229 | Uslada | FAM (A) | HEX (A) | HEX (C) | HEX | FAM (C) | *vrn-A1b* | vrn-A1 Winter | *vrn-B1* (winter) | A | T | C |
| 230 | Zoloto Dona (993/ 12) | FAM (A) | HEX (A) | HEX (C) | HEX | HEX (G) | *vrn-A1b* | vrn-A1 Winter | *vrn-B1* (winter) | G | T | A |
| 231 | 796/11 | FAM (A) | HEX (A) | HEX (C) | HEX | FAM (C) | Vrn-A1a | vrn-A1 Winter | *vrn-B1* (winter) | A | T | C |
| 232 | 589/13 | FAM (A) | HEX (A) | HEX (C) | HEX | FAM (C) | *vrn-A1b* | vrn-A1 Winter | *vrn-B1* (winter) | G | T | A |
| 233 | 737/11 | FAM (A) | HEX (A) | HEX (C) | HEX | heterogeneous | *vrn-A1b* | vrn-A1 Winter | *vrn-B1* (winter) | G | T | C |
| 234 | 655/13 (Solntsedar) | FAM (A) | HEX (A) | HEX (C) | HEX | heterogeneous | *vrn-A1b* | vrn-A1 Winter | *vrn-B1* (winter) | G | T | A |
| 235 | 533/14 | FAM (A) | HEX (A) | HEX (C) | HEX | heterogeneous | *vrn-A1b* | vrn-A1 Winter | *vrn-B1* (winter) | G | T | C |
| 236 | Prikumchanka | FAM (A) | HEX (A) | HEX (C) | HEX | FAM (C) | *vrn-A1b* | vrn-A1 Winter | *vrn-B1* (winter) | G | T | A |
| 237 | Stepnoy yantar | FAM (A) | HEX (A) | HEX (C) | HEX | FAM (C) | *vrn-A1b* | vrn-A1 Winter | *vrn-B1* (winter) | A | T | C |
| 238 | Zhivitsa | FAM (A) | HEX (A) | HEX (C) | HEX | FAM (C) | *vrn-A1b* | vrn-A1 Winter | *vrn-B1* (winter) | A | T | A |
| 239 | Bagryanitsa (Italikum) | FAM (A) | HEX (A) | HEX (C) | HEX | HEX (G) | *vrn-A1b* | vrn-A1 Winter | *vrn-B1* (winter) | G | T | C |
| 240 | Perlina odesskaya | FAM (A) | HEX (A) | HEX (C) | HEX | FAM (C) | *vrn-A1b* | vrn-A1 Winter | *vrn-B1* (winter) | G | T | A |
| 241 | Burshtyn | FAM (A) | HEX (A) | HEX (C) | HEX | FAM (C) | *vrn-A1b* | vrn-A1 Winter | *vrn-B1* (winter) | G | C | A |
| 242 | Kontinent | FAM (A) | HEX (A) | HEX (C) | HEX | FAM (C) | *vrn-A1b* | vrn-A1 Winter | *vrn-B1* (winter) | G | T | A |
| 243 | Akveduk | FAM (A) | HEX (A) | HEX (C) | HEX | FAM (C) | *vrn-A1b* | vrn-A1 Winter | *vrn-B1* (winter) | G | T | A |
| 244 | Andromeda | FAM (A) | HEX (A) | HEX (C) | HEX | FAM (C) | *vrn-A1b* | vrn-A1 Winter | *vrn-B1* (winter) | G | T | A |
| 245 | Dnepryana | FAM (A) | HEX (A) | HEX (C) | heterogeneous | FAM (C) | *vrn-A1b* | vrn-A1 Winter | *vrn-B1* (winter) | G | T | A |
| 246 | Kharkovskaya 32 | FAM (A) | HEX (A) | HEX (C) | HEX | heterogeneous | *vrn-A1b* | vrn-A1 Winter | *vrn-B1* (winter) | G | T | A |
| 247 | Leukurum 36 | HEX (G) | FAM (G) | HEX (C) | FAM | HEX (G) | *vrn-A1b* | vrn-A1 Winter | *vrn-B1* (winter) | G | T | A |
| 248 | Afina | FAM (A) | HEX (A) | HEX (C) | HEX | FAM (C) | *vrn-A1b* | vrn-A1 Winter | *vrn-B1* (winter) | G | T | A |
| 249 | Makar | FAM (A) | HEX (A) | HEX (C) | HEX | FAM (C) | *vrn-A1b* | vrn-A1 Winter | *vrn-B1* (winter) | G | T | A |
| 250 | Belgorodskaya yantarnaya | FAM (A) | HEX (A) | HEX (C) | HEX | FAM (C) | *vrn-A1b* | vrn-A1 Winter | *vrn-B1* (winter) | G | T | A |
| 251 | Temnyy yantar | FAM (A) | HEX (A) | HEX (C) | HEX | FAM (C) | *vrn-A1b* | vrn-A1 Winter | *vrn-B1* (winter) | G | T | A |
| 252 | Koshelevskaya | FAM (A) | HEX (A) | HEX (C) | HEX | heterogeneous | *vrn-A1b* | vrn-A1 Winter | *vrn-B1* (winter) | G | T | A |
| 253 | Shulyndinka | FAM (A) | HEX (A) | HEX (C) | HEX | FAM (C) | *vrn-A1b* | vrn-A1 Winter | *vrn-B1* (winter) | G | T | A |
| 254 | Pributkova | FAM (A) | HEX (A) | HEX (C) | HEX | FAM (C) | *vrn-A1b* | vrn-A1 Winter | *vrn-B1* (winter) | G | T | A |
| 255 | Yagut | HEX (G) | HEX (A) | HEX (C) | FAM | FAM (C) | *vrn-A1b* | vrn-A1 Winter | *vrn-B1* (winter) | A | T | A |
| 256 | DF -104/85 | FAM (A) | HEX (A) | HEX (C) | HEX | FAM (C) | *vrn-A1b* | vrn-A1 Winter | *vrn-B1* (winter) | G | T | A |
| 257 | Pandur | FAM (A) | HEX (A) | HEX (C) | HEX | heterogeneous | *vrn-A1b* | vrn-A1 Winter | *vrn-B1* (winter) | G | T | A |
| 258 | Elidur | FAM (A) | HEX (A) | HEX (C) | HEX | heterogeneous | *vrn-A1b* | vrn-A1 Winter | *vrn-B1* (winter) | A | C | C |
| 259 | Rodur | HEX (G) | FAM (G) | FAM (G) | FAM | heterogeneous | Vrn-A1a | vrn-A1 Winter | *vrn-B1* (winter) | G | T | A |
| 260 | Condur | FAM (A) | HEX (A) | HEX (C) | HEX | FAM (C) | Vrn-A1a | vrn-A1 Winter | *vrn-B1* (winter) | A | T | A |
| 261 | Blindur/DF 38-86/3 | FAM (A) | HEX (A) | HEX (C) | HEX | FAM (C) | *vrn-A1b* | vrn-A1 Winter | *vrn-B1* (winter) | G | C | C |
| 262 | Rumyniya DF-01058-1-101 | FAM (A) | HEX (A) | HEX (C) | HEX | FAM (C) | *vrn-A1b* | vrn-A1 Winter | *vrn-B1* (winter) | A | C | C |
| 263 | Rumyniya DF-00091-14-002 | FAM (A) | HEX (A) | HEX (C) | HEX | FAM (C) | Vrn-A1a | vrn-A1 Winter | *vrn-B1* (winter) | G | T | A |
| 264 | Martondur 1 | FAM (A) | HEX (A) | HEX (C) | FAM | FAM (C) | Vrn-A1a | vrn-A1 Winter | *vrn-B1* (winter) | A | C | A |
| 265 | MVTD 12-97 | FAM (A) | HEX (A) | HEX (C) | HEX | FAM (C) | *vrn-A1b* | vrn-A1 Winter | *vrn-B1* (winter) | G | T | A |
| 266 | MVTD 12-99 | FAM (A) | HEX (A) | HEX (C) | FAM | HEX (G) | *vrn-A1b* | vrn-A1 Winter | *vrn-B1* (winter) | G | T | A |
| 267 | MVTD 15-98 | FAM (A) | HEX (A) | HEX (C) | HEX | HEX (G) | *vrn-A1b* | vrn-A1 Winter | *vrn-B1* (winter) | G | T | A |
| 268 | MV Makaroni | FAM (A) | HEX (A) | HEX (C) | HEX | FAM (C) | *vrn-A1b* | vrn-A1 Winter | *vrn-B1* (winter) | G | T | A |
| 269 | MV HUNDUR | HEX (G) | FAM (G) | heterogeneous | FAM | HEX (G) | *vrn-A1b* | vrn-A1 Winter | *vrn-B1* (winter) | G | T | A |
| 270 | MV PENNEDUR | HEX (G) | FAM (G) | FAM (G) | FAM | HEX (G) | *vrn-A1b* | vrn-A1 Winter | *vrn-B1* (winter) | G | T | A |
| 271 | Wintergold | FAM (A) | HEX (A) | HEX (C) | HEX | FAM (C) | Vrn-A1a | vrn-A1 Winter | *vrn-B1* (winter) | G | C | C |
| 272 | AMA 11855t85-90 | HEX (G) | FAM (G) | FAM (G) | FAM | FAM (C) | Vrn-A1a | vrn-A1 Winter | *vrn-B1* (winter) | G | C | C |
| 273 | AMA 15908ds1122-07 | HEX (G) | FAM (G) | FAM (G) | FAM | heterogeneous | Vrn-A1a | vrn-A1 Winter | *vrn-B1* (winter) | G | C | C |
| 274 | 21545t | HEX (G) | HEX (A) | FAM (G) | FAM | HEX (G) | Vrn-A1a | vrn-A1 Winter | *vrn-B1* (winter) | A | C | C |
| 275 | 3561h28 | FAM (A) | HEX (A) | HEX (C) | HEX | FAM (C) | *vrn-A1b* | vrn-A1 Winter | *vrn-B1* (winter) | G | T | A |
| 276 | 3622h46 | FAM (A) | HEX (A) | HEX (C) | HEX | HEX (G) | *vrn-A1b* | vrn-A1 Winter | *vrn-B1* (winter) | G | C | C |
| 277 | 3591h96 | FAM (A) | HEX (A) | HEX (C) | HEX | heterogeneous | *vrn-A1b* | vrn-A1 Winter | *vrn-B1* (winter) | G | T | A |
| 278 | 3336h6-13-162 | HEX (G) | HEX (A) | FAM (G) | FAM | FAM (C) | *vrn-A1b* | vrn-A1 Winter | *vrn-B1* (winter) | G | T | C |
| 279 | 3680h62 (Belka) | FAM (A) | HEX (A) | HEX (C) | HEX | FAM (C) | *vrn-A1b* | vrn-A1 Winter | *vrn-B1* (winter) | G | C | C |
| 280 | 3796h33 | FAM (A) | HEX (A) | HEX (C) | HEX | heterogeneous | *vrn-A1b* | vrn-A1 Winter | *vrn-B1* (winter) | G | T | A |
| 326 | 3850h69 (Tsel) | FAM (A) | HEX (A) | HEX (C) | HEX | HEX (G) | *vrn-A1b* | vrn-A1 Winter | *vrn-B1* (winter) | G | T | A |
| 327 | 3527h4-14-3 | FAM (A) | HEX (A) | HEX (C) | HEX | heterogeneous | Vrn-A1a | vrn-A1 Winter | *vrn-B1* (winter) | G | T | A |
| 328 | 3609h36-14-4 (Bella) | FAM (A) | HEX (A) | HEX (C) | HEX | HEX (G) | *vrn-A1b* | vrn-A1 Winter | *vrn-B1* (winter) | G | C | A |
| 329 | 3982h42 | FAM (A) | HEX (A) | HEX (C) | HEX | heterogeneous | Vrn-A1a | vrn-A1 Winter | *vrn-B1* (winter) | G | T | C |
| 330 | 3359h18-15-174 | FAM (A) | HEX (A) | HEX (C) | HEX | HEX (G) | *vrn-A1b* | vrn-A1 Winter | *vrn-B1* (winter) | G | heterogeneous | C |
| 331 | 3896h145 | FAM (A) | HEX (A) | HEX (C) | HEX | HEX (G) | *vrn-A1b* | vrn-A1 Winter | *vrn-B1* (winter) | G | C | C |
| 332 | 3934h148 | FAM (A) | HEX (A) | HEX (C) | HEX | HEX (G) | *vrn-A1b* | vrn-A1 Winter | *vrn-B1* (winter) | G | T | A |
| 333 | 3934h155 | FAM (A) | HEX (A) | HEX (C) | HEX | HEX (G) | *vrn-A1b* | vrn-A1 Winter | *vrn-B1* (winter) | G | T | A |
| 334 | 4089h94 | FAM (A) | HEX (A) | HEX (C) | HEX | heterogeneous | *vrn-A1b* | vrn-A1 Winter | *vrn-B1* (winter) | G | T | A |
| 335 | 4135h7 | FAM (A) | HEX (A) | HEX (C) | HEX | HEX (G) | *vrn-A1b* | vrn-A1 Winter | *vrn-B1* (winter) | G | T | C |
| 336 | 4218h77 | FAM (A) | HEX (A) | HEX (C) | HEX | HEX (G) | *vrn-A1b* | vrn-A1 Winter | *vrn-B1* (winter) | G | T | A |
| 337 | 4169h75 | FAM (A) | HEX (A) | HEX (C) | HEX | HEX (G) | *vrn-A1b* | vrn-A1 Winter | *vrn-B1* (winter) | G | C | C |
| 338 | 4192h94 | FAM (A) | HEX (A) | HEX (C) | HEX | HEX (G) | *vrn-A1b* | vrn-A1 Winter | *vrn-B1* (winter) | G | C | C |
| 339 | 4192h98 | FAM (A) | HEX (A) | HEX (C) | HEX | HEX (G) | *vrn-A1b* | vrn-A1 Winter | *vrn-B1* (winter) | G | C | A |
| 340 | 4336h7 (Zashchita) | FAM (A) | HEX (A) | HEX (C) | HEX | HEX (G) | *vrn-A1b* | vrn-A1 Winter | *vrn-B1* (winter) | G | C | A |
| 341 | 4336h24 | FAM (A) | HEX (A) | HEX (C) | HEX | HEX (G) | *vrn-A1b* | vrn-A1 Winter | *vrn-B1* (winter) | G | C | C |
| 342 | 4337h3 | FAM (A) | HEX (A) | HEX (C) | HEX | heterogeneous | *vrn-A1b* | vrn-A1 Winter | *vrn-B1* (winter) | G | C | C |
| 343 | 4341h1 | FAM (A) | HEX (A) | HEX (C) | HEX | HEX (G) | *vrn-A1b* | vrn-A1 Winter | *vrn-B1* (winter) | G | C | C |
| 344 | 4342h23 | FAM (A) | HEX (A) | HEX (C) | HEX | HEX (G) | *vrn-A1b* | vrn-A1 Winter | *vrn-B1* (winter) | G | C | C |
| 345 | 3596h56-15-22 (Dara) | FAM (A) | HEX (A) | HEX (C) | HEX | HEX (G) | *vrn-A1b* | vrn-A1 Winter | *vrn-B1* (winter) | G | C | A |
| 346 | 3596h56-15-69 | FAM (A) | HEX (A) | HEX (C) | HEX | heterogeneous | *vrn-A1b* | vrn-A1 Winter | *vrn-B1* (winter) | G | C | A |
| 347 | 4306h15 | FAM (A) | HEX (A) | HEX (C) | HEX | HEX (G) | *vrn-A1b* | vrn-A1 Winter | *vrn-B1* (winter) | G | C | A |
| 348 | 4320h38 | FAM (A) | HEX (A) | HEX (C) | HEX | HEX (G) | *vrn-A1b* | vrn-A1 Winter | *vrn-B1* (winter) | G | C | A |
| 349 | 4341h68 | FAM (A) | HEX (A) | HEX (C) | HEX | HEX (G) | *vrn-A1b* | vrn-A1 Winter | *vrn-B1* (winter) | G | C | A |
| 350 | 4353h47 | FAM (A) | HEX (A) | HEX (C) | HEX | HEX (G) | *vrn-A1b* | vrn-A1 Winter | *vrn-B1* (winter) | G | C | C |
| 351 | 4383h72 | FAM (A) | HEX (A) | HEX (C) | HEX | heterogeneous | *vrn-A1b* | vrn-A1 Winter | *vrn-B1* (winter) | A | T | C |
| 352 | 4387h148 | FAM (A) | HEX (A) | HEX (C) | HEX | FAM (C) | *vrn-A1b* | vrn-A1 Winter | *vrn-B1* (winter) | G | T | A |
| 353 | 4392h17 | FAM (A) | HEX (A) | HEX (C) | HEX | FAM (C) | *vrn-A1b* | vrn-A1 Winter | *vrn-B1* (winter) | A | T | C |
| 354 | 4416h49 skorosp. | FAM (A) | HEX (A) | HEX (C) | HEX | FAM (C) | *vrn-A1b* | vrn-A1 Winter | *vrn-B1* (winter) | G | C | A |
| 355 | 4419h45 | FAM (A) | HEX (A) | HEX (C) | HEX | FAM (C) | Vrn-A1a | vrn-A1 Winter | *vrn-B1* (winter) | G | T | A |
| 356 | 4424h4 | FAM (A) | HEX (A) | HEX (C) | HEX | HEX (G) | *vrn-A1b* | vrn-A1 Winter | *vrn-B1* (winter) | heterogeneous | T | A |
| 357 | 4424h96 | FAM (A) | HEX (A) | HEX (C) | HEX | heterogeneous | *vrn-A1b* | vrn-A1 Winter | *vrn-B1* (winter) | G | T | A |
| 358 | 4428h110 | FAM (A) | HEX (A) | HEX (C) | HEX | HEX (G) | *vrn-A1b* | vrn-A1 Winter | *vrn-B1* (winter) | G | T | A |
| 359 | 4430h50 | FAM (A) | HEX (A) | HEX (C) | HEX | HEX (G) | *vrn-A1b* | vrn-A1 Winter | *vrn-B1* (winter) | G | C | C |
| 360 | 4432h24 | FAM (A) | HEX (A) | HEX (C) | HEX | FAM (C) | *vrn-A1b* | vrn-A1 Winter | *vrn-B1* (winter) | G | T | C |
| 361 | 4432h45 | FAM (A) | HEX (A) | HEX (C) | HEX | FAM (C) | *vrn-A1b* | vrn-A1 Winter | *vrn-B1* (winter) | A | T | C |
| 362 | 4433h52 | FAM (A) | HEX (A) | HEX (C) | HEX | FAM (C) | *vrn-A1b* | vrn-A1 Winter | *vrn-B1* (winter) | A | T | C |
| 363 | 4433h90 | FAM (A) | HEX (A) | HEX (C) | HEX | heterogeneous | *vrn-A1b* | vrn-A1 Winter | *vrn-B1* (winter) | heterogeneous | T | C |
| 364 | 4466h183 | FAM (A) | HEX (A) | HEX (C) | HEX | FAM (C) | *vrn-A1b* | vrn-A1 Winter | *vrn-B1* (winter) | G | T | C |
| 365 | 4467h18 | FAM (A) | HEX (A) | HEX (C) | HEX | heterogeneous | *vrn-A1b* | vrn-A1 Winter | *vrn-B1* (winter) | G | C | C |
| 366 | 2468h10-06M-17-10 | FAM (A) | HEX (A) | HEX (C) | HEX | HEX (G) | *vrn-A1b* | vrn-A1 Winter | *vrn-B1* (winter) | G | T | A |
| 367 | 3336h43-17-128 | FAM (A) | HEX (A) | HEX (C) | HEX | heterogeneous | *vrn-A1b* | vrn-A1 Winter | *vrn-B1* (winter) | G | T | A |
| 368 | 3903h9-17-3 | FAM (A) | HEX (A) | HEX (C) | HEX | heterogeneous | *vrn-A1b* | vrn-A1 Winter | *vrn-B1* (winter) | G | T | A |
| 369 | 4167h48-17-12 | FAM (A) | HEX (A) | HEX (C) | HEX | heterogeneous | *vrn-A1b* | vrn-A1 Winter | *vrn-B1* (winter) | G | T | A |
| 370 | 4250hK-19 | FAM (A) | HEX (A) | HEX (C) | HEX | HEX (G) | Vrn-A1a | vrn-A1 Winter | *vrn-B1* (winter) | G | T | A |
| 371 | 3902h3-18-31 | FAM (A) | HEX (A) | HEX (C) | HEX | heterogeneous | *vrn-A1b* | vrn-A1 Winter | *vrn-B1* (winter) | A | T | C |
| 372 | 3914h38-18-8 | FAM (A) | HEX (A) | HEX (C) | HEX | heterogeneous | *vrn-A1b* | vrn-A1 Winter | *vrn-B1* (winter) | G | T | C |
| 373 | 4477h2 | FAM (A) | HEX (A) | HEX (C) | HEX | HEX (G) | *vrn-A1b* | vrn-A1 Winter | *vrn-B1* (winter) | G | C | C |
| 374 | 4482h8 | FAM (A) | HEX (A) | HEX (C) | HEX | heterogeneous | *vrn-A1b* | vrn-A1 Winter | *vrn-B1* (winter) | A | T | C |
| 375 | 4505h38 | FAM (A) | HEX (A) | HEX (C) | HEX | FAM (C) | Vrn-A1a | vrn-A1 Winter | *vrn-B1* (winter) | A | C | C |
| 376 | 4509h13 | FAM (A) | HEX (A) | HEX (C) | HEX | FAM (C) | *vrn-A1b* | vrn-A1 Winter | *vrn-B1* (winter) | G | T | A |
| 377 | 4517h25 | FAM (A) | HEX (A) | HEX (C) | HEX | FAM (C) | *vrn-A1b* | vrn-A1 Winter | *vrn-B1* (winter) | A | C | C |
| 378 | 4521h13 | FAM (A) | HEX (A) | HEX (C) | HEX | HEX (G) | *vrn-A1b* | vrn-A1 Winter | *vrn-B1* (winter) | G | T | C |
| 379 | 4574h8 | FAM (A) | HEX (A) | HEX (C) | HEX | heterogeneous | *vrn-A1b* | vrn-A1 Winter | *vrn-B1* (winter) | A | T | C |
| 380 | 4619h9 | FAM (A) | HEX (A) | HEX (C) | HEX | HEX (G) | *vrn-A1b* | vrn-A1 Winter | *vrn-B1* (winter) | G | C | A |
| 381 | 4619h34 | FAM (A) | HEX (A) | HEX (C) | HEX | HEX (G) | *vrn-A1b* | vrn-A1 Winter | *vrn-B1* (winter) | G | C | C |
| 382 | 4626h15 | FAM (A) | HEX (A) | HEX (C) | HEX | HEX (G) | *vrn-A1b* | vrn-A1 Winter | *vrn-B1* (winter) | heterogeneous | C | A |
| 383 | 4631h14 | FAM (A) | HEX (A) | HEX (C) | HEX | HEX (G) | *vrn-A1b* | vrn-A1 Winter | *vrn-B1* (winter) | heterogeneous | C | A |
| 384 | 4652h45 | FAM (A) | HEX (A) | HEX (C) | HEX | FAM (C) | Vrn-A1a | vrn-A1 Winter | *vrn-B1* (winter) | A | T | A |
| 385 | 4681h53 | FAM (A) | HEX (A) | HEX (C) | HEX | FAM (C) | Vrn-A1a | vrn-A1 Winter | *vrn-B1* (winter) | heterogeneous | T | C |
| 386 | 4686h35 | FAM (A) | HEX (A) | HEX (C) | HEX | heterogeneous | *vrn-A1b* | vrn-A1 Winter | *vrn-B1* (winter) | G | C | A |
| 387 | 771p46-18-1 | FAM (A) | HEX (A) | HEX (C) | HEX | HEX (G) | *vrn-A1b* | vrn-A1 Winter | *vrn-B1* (winter) | G | C | A |
| 539 | 4519h24 | FAM (A) | HEX (A) | HEX (C) | HEX | HEX (G) | *vrn-A1b* | vrn-A1 Winter | *vrn-B1* (winter) | G | C | A |
| 540 | 4528h1 | FAM (A) | HEX (A) | HEX (C) | HEX | HEX (G) | *vrn-A1b* | vrn-A1 Winter | *vrn-B1* (winter) | G | T | A |
| 541 | 4529h25 | FAM (A) | HEX (A) | HEX (C) | HEX | FAM (C) | *vrn-A1b* | vrn-A1 Winter | *vrn-B1* (winter) | G | T | A |
| 542 | 4557h38 | FAM (A) | HEX (A) | HEX (C) | HEX | HEX (G) | *vrn-A1b* | vrn-A1 Winter | *vrn-B1* (winter) | A | T | A |
| 543 | 4626h23 | FAM (A) | HEX (A) | HEX (C) | HEX | FAM (C) | *vrn-A1b* | vrn-A1 Winter | *vrn-B1* (winter) | G | T | A |
| 544 | 4639h102 | FAM (A) | HEX (A) | HEX (C) | HEX | FAM (C) | *vrn-A1b* | vrn-A1 Winter | *vrn-B1* (winter) | A | T | C |
| 545 | 4643h29 | FAM (A) | HEX (A) | HEX (C) | HEX | HEX (G) | *vrn-A1b* | vrn-A1 Winter | *vrn-B1* (winter) | A | T | C |
| 546 | 4646h1 | FAM (A) | HEX (A) | HEX (C) | HEX | FAM (C) | *vrn-A1b* | vrn-A1 Winter | *vrn-B1* (winter) | G | T | A |
| 547 | 4675h11 | FAM (A) | HEX (A) | HEX (C) | HEX | heterogeneous | *vrn-A1b* | vrn-A1 Winter | *vrn-B1* (winter) | G | heterogeneous | C |
| 548 | 4518h20 | FAM (A) | HEX (A) | HEX (C) | HEX | FAM (C) | *vrn-A1b* | vrn-A1 Winter | *vrn-B1* (winter) | G | T | C |
| 549 | 3902h3-18-3 | FAM (A) | HEX (A) | HEX (C) | HEX | FAM (C) | *vrn-A1b* | vrn-A1 Winter | *vrn-B1* (winter) | A | T | C |
| 550 | 4822h19 | FAM (A) | HEX (A) | HEX (C) | HEX | HEX (G) | *vrn-A1b* | vrn-A1 Winter | *vrn-B1* (winter) | G | T | A |
| 551 | 3408h218-18-173 | FAM (A) | HEX (A) | HEX (C) | HEX | FAM (C) | *vrn-A1b* | vrn-A1 Winter | *vrn-B1* (winter) | G | C | A |
| 552 | 3683h46-18-59 | FAM (A) | HEX (A) | HEX (C) | HEX | heterogeneous | *vrn-A1b* | vrn-A1 Winter | *vrn-B1* (winter) | G | T | C |
| 554 | 4428h48-19-11 | FAM (A) | HEX (A) | HEX (C) | HEX | heterogeneous | Vrn-A1a | vrn-A1 Winter | *vrn-B1* (winter) | G | T | A |
| 555 | 4778h29 | FAM (A) | HEX (A) | HEX (C) | HEX | HEX (G) | *vrn-A1b* | vrn-A1 Winter | *vrn-B1* (winter) | heterogeneous | T | A |
| 556 | 4779h57 | FAM (A) | HEX (A) | HEX (C) | HEX | FAM (C) | *vrn-A1b* | vrn-A1 Winter | *vrn-B1* (winter) | G | C | A |
| 557 | 4812h7 | FAM (A) | HEX (A) | HEX (C) | HEX | HEX (G) | *vrn-A1b* | vrn-A1 Winter | *vrn-B1* (winter) | G | T | A |
| 560 | 4626h10-20-24 | FAM (A) | HEX (A) | HEX (C) | HEX | FAM (C) | Vrn-A1a | vrn-A1 Winter | *vrn-B1* (winter) | A | C | C |

Note to the *VRN-A1* column: *vrn-A1a*, Jagger type (short vernalization); *vrn-A1b*, 2147 type (long vernalization).

Table S2. Phenotypes of the studied collection: frost tolerance, spring regrowth, overwintering (per-accession means).

| **sample_id** | **Cultivar / line** | **Frost tolerance, %** | **Spring regrowth, score** | **Overwintering, score** | **n obs. (frost tol.)** |
| --- | --- | --- | --- | --- | --- |
| 20 | Krupinka | 52.4 | 4.8 | 6.0 | 3 |
| 40 | Krucha | 34.3 | 7.7 | 7.0 | 3 |
| 203 | Kristall 2 | 2.4 | 6.3 | 7.0 | 3 |
| 204 | Leukurum 21 | 30.1 | 8.0 | 8.0 | 3 |
| 205 | Alena | 28.3 | 8.3 | 8.0 | 3 |
| 206 | Kermen | 34.3 | 8.0 | 7.0 | 3 |
| 207 | Uniya | 36.2 | 7.3 | 8.0 | 3 |
| 208 | Zolotko | 34.4 | 7.0 | 8.0 | 3 |
| 209 | Laska | 37.3 | 7.7 | 8.0 | 3 |
| 210 | Odari | 54.7 | 5.0 | 6.0 | 3 |
| 211 | Altana | 47.4 | 5.3 | 7.0 | 3 |
| 212 | Kordon | 59.2 | 5.3 | 6.0 | 3 |
| 213 | Dobryana | 20.3 | 8.0 | 8.3 | 3 |
| 214 | Sinora | 50.3 | 7.5 | 9.0 | 11 |
| 215 | Donchanka | 45.6 | 6.3 | 7.0 | 3 |
| 216 | Kurant | 27.7 | 7.3 | 7.0 | 3 |
| 217 | Kristella | 34.7 | 7.0 | 7.0 | 3 |
| 218 | Amazonka | 61.9 | 5.3 | 7.0 | 3 |
| 219 | Agat donskoy | 66.7 | 7.0 | 8.0 | 3 |
| 220 | Lazurit | 50.2 | 6.3 | 8.0 | 3 |
| 221 | Oniks | 56.2 | 6.3 | 8.0 | 3 |
| 222 | Diona | 36.6 | 7.7 | 7.0 | 3 |
| 223 | Kiprida | 31.7 | 6.7 | 7.0 | 3 |
| 224 | Teyya | 52.8 | 6.3 | 7.0 | 3 |
| 225 | Eyrena | 55.1 | 6.0 | 7.0 | 3 |
| 226 | Yakhont (760/10) | 26.7 | 6.0 | 6.0 | 3 |
| 227 | Yubilyarka (330/10) | 66.6 | 6.0 | 8.0 | 3 |
| 228 | Yantarina | 56.3 | 5.7 |  | 3 |
| 229 | Uslada | 57.0 | 7.0 |  | 3 |
| 230 | Zoloto Dona (993/ 12) | 40.3 | 6.7 | 7.3 | 3 |
| 231 | 796/11 | 59.7 | 6.3 | 8.0 | 3 |
| 232 | 589/13 | 54.3 | 5.7 | 8.0 | 3 |
| 233 | 737/11 | 19.6 | 7.7 |  | 3 |
| 234 | 655/13 (Solntsedar) | 37.1 | 6.7 |  | 3 |
| 235 | 533/14 | 36.8 | 7.0 |  | 3 |
| 236 | Prikumchanka | 35.7 | 7.7 | 8.0 | 3 |
| 237 | Stepnoy yantar | 47.2 | 7.3 | 6.0 | 3 |
| 238 | Zhivitsa | 15.6 | 8.3 | 8.0 | 3 |
| 239 | Bagryanitsa (Italikum) | 49.1 | 5.3 | 6.0 | 3 |
| 240 | Perlina odesskaya | 34.3 | 7.0 | 8.0 | 3 |
| 241 | Burshtyn | 36.7 | 7.3 | 8.0 | 3 |
| 242 | Kontinent | 20.3 | 7.0 | 7.0 | 3 |
| 243 | Akveduk | 25.4 | 7.0 | 8.0 | 3 |
| 244 | Andromeda | 22.9 | 7.7 | 8.0 | 3 |
| 245 | Dnepryana | 16.2 | 7.0 | 8.3 | 3 |
| 246 | Kharkovskaya 32 | 36.3 | 8.3 | 7.0 | 3 |
| 247 | Leukurum 36 | 34.8 | 6.0 | 7.0 | 3 |
| 248 | Afina | 39.6 | 6.3 | 6.0 | 3 |
| 249 | Makar | 46.8 | 6.0 | 8.0 | 3 |
| 250 | Belgorodskaya yantarnaya | 63.4 | 6.0 | 8.0 | 3 |
| 251 | Temnyy yantar | 74.9 | 5.0 |  | 3 |
| 252 | Koshelevskaya | 36.3 | 6.0 |  | 3 |
| 253 | Shulyndinka | 36.6 | 6.0 | 8.0 | 3 |
| 254 | Pributkova | 33.2 | 5.3 | 8.0 | 3 |
| 255 | Yagut | 0.0 | 7.3 | 8.0 | 3 |
| 256 | DF -104/85 | 39.4 | 8.7 | 8.0 | 3 |
| 257 | Pandur | 54.6 | 9.1 | 7.0 | 3 |
| 258 | Elidur | 33.5 | 8.8 | 6.0 | 3 |
| 259 | Rodur | 1.3 | 8.3 | 8.0 | 3 |
| 260 | Condur | 19.5 | 8.3 | 8.0 | 3 |
| 261 | Blindur/DF 38-86/3 | 25.9 | 8.0 | 7.0 | 3 |
| 262 | Rumyniya DF-01058-1-101 | 8.7 | 8.7 | 7.0 | 3 |
| 263 | Rumyniya DF-00091-14-002 | 20.7 | 7.7 | 6.0 | 3 |
| 264 | Martondur 1 | 1.3 | 8.3 | 8.0 | 3 |
| 265 | MVTD 12-97 | 19.3 | 8.0 | 7.0 | 3 |
| 266 | MVTD 12-99 | 8.0 | 8.3 | 7.0 | 3 |
| 267 | MVTD 15-98 | 47.9 | 7.3 | 7.0 | 3 |
| 268 | MV Makaroni | 15.6 | 6.0 | 8.0 | 3 |
| 269 | MV HUNDUR | 2.7 | 7.7 | 8.0 | 3 |
| 270 | MV PENNEDUR | 5.0 | 6.7 | 7.0 | 3 |
| 271 | Wintergold | 1.7 | 8.7 | 6.0 | 3 |
| 272 | AMA 11855t85-90 | 5.3 | 9.4 | 6.0 | 3 |
| 273 | AMA 15908ds1122-07 | 4.3 | 9.4 | 6.0 | 3 |
| 274 | 21545t | 3.3 | 6.7 | 7.0 | 3 |
| 275 | 3561h28 | 30.7 | 5.0 |  | 3 |
| 276 | 3622h46 | 41.6 | 7.0 |  | 9 |
| 277 | 3591h96 | 6.1 | 6.0 |  | 3 |
| 278 | 3336h6-13-162 | 9.0 | 5.0 |  | 3 |
| 279 | 3680h62 (Belka) | 56.0 | 6.6 |  | 12 |
| 280 | 3796h33 | 45.3 | 4.0 |  | 3 |
| 326 | 3850h69 (Tsel) | 65.9 | 6.2 |  | 12 |
| 327 | 3527h4-14-3 | 58.1 | 4.8 |  | 9 |
| 328 | 3609h36-14-4 (Bella) | 49.8 | 5.8 |  | 12 |
| 329 | 3982h42 | 61.2 | 5.2 |  | 9 |
| 330 | 3359h18-15-174 | 63.7 | 4.2 |  | 9 |
| 331 | 3896h145 | 58.4 | 4.0 |  | 9 |
| 332 | 3934h148 | 28.4 | 6.4 |  | 11 |
| 333 | 3934h155 | 55.5 | 5.0 |  | 9 |
| 334 | 4089h94 | 39.1 | 6.3 |  | 12 |
| 335 | 4135h7 | 34.7 | 6.8 |  | 11 |
| 336 | 4218h77 | 62.9 | 6.4 |  | 8 |
| 337 | 4169h75 | 27.5 | 7.0 |  | 6 |
| 338 | 4192h94 | 33.7 | 6.2 |  | 6 |
| 339 | 4192h98 | 65.5 | 5.5 |  | 6 |
| 340 | 4336h7 (Zashchita) | 41.8 | 5.2 |  | 12 |
| 341 | 4336h24 | 49.4 | 4.8 |  | 12 |
| 342 | 4337h3 | 46.4 | 5.4 |  | 12 |
| 343 | 4341h1 | 40.9 | 4.5 |  | 9 |
| 344 | 4342h23 | 52.1 | 5.8 |  | 6 |
| 345 | 3596h56-15-22 (Dara) | 41.8 | 5.0 |  | 11 |
| 346 | 3596h56-15-69 | 55.0 | 4.5 |  | 11 |
| 347 | 4306h15 | 57.1 | 6.0 |  | 8 |
| 348 | 4320h38 | 47.1 | 6.0 |  | 5 |
| 349 | 4341h68 | 42.3 | 5.5 |  | 5 |
| 350 | 4353h47 | 57.3 | 9.0 |  | 5 |
| 351 | 4383h72 | 68.5 | 6.7 |  | 6 |
| 352 | 4387h148 | 46.5 | 8.0 |  | 5 |
| 353 | 4392h17 | 59.8 | 7.5 |  | 5 |
| 354 | 4416h49 skorosp. | 54.1 | 5.5 |  | 5 |
| 355 | 4419h45 | 54.0 | 6.0 |  | 5 |
| 356 | 4424h4 | 46.5 | 8.0 |  | 5 |
| 357 | 4424h96 | 59.8 | 6.3 |  | 6 |
| 358 | 4428h110 | 45.8 | 7.0 |  | 5 |
| 359 | 4430h50 | 32.7 | 7.0 |  | 5 |
| 360 | 4432h24 | 51.7 | 6.5 |  | 5 |
| 361 | 4432h45 | 33.4 | 7.5 |  | 5 |
| 362 | 4433h52 | 49.1 | 6.5 |  | 5 |
| 363 | 4433h90 | 65.2 | 7.5 |  | 5 |
| 364 | 4466h183 | 73.1 | 6.5 |  | 5 |
| 365 | 4467h18 | 58.2 | 7.0 |  | 5 |
| 366 | 2468h10-06M-17-10 | 60.3 | 6.7 |  | 9 |
| 367 | 3336h43-17-128 | 66.0 | 7.0 |  | 8 |
| 368 | 3903h9-17-3 | 54.5 | 7.0 |  | 8 |
| 369 | 4167h48-17-12 | 52.1 | 7.5 |  | 9 |
| 370 | 4250hK-19 | 63.9 | 5.5 |  | 5 |
| 371 | 3902h3-18-31 | 82.8 | 6.5 | 9.0 | 5 |
| 372 | 3914h38-18-8 | 55.8 | 7.0 | 9.0 | 8 |
| 373 | 4477h2 | 64.0 | 5.5 | 9.0 | 5 |
| 374 | 4482h8 | 42.1 | 7.0 | 7.3 | 5 |
| 375 | 4505h38 | 64.9 | 7.0 | 9.0 | 4 |
| 376 | 4509h13 | 51.9 | 7.0 | 9.0 | 8 |
| 377 | 4517h25 | 75.3 | 8.0 | 8.0 | 4 |
| 378 | 4521h13 | 66.5 | 7.0 | 9.0 | 8 |
| 379 | 4574h8 | 17.9 | 9.0 | 9.0 | 7 |
| 380 | 4619h9 | 39.8 | 7.0 | 9.0 | 7 |
| 381 | 4619h34 | 54.3 | 7.0 | 9.0 | 4 |
| 382 | 4626h15 | 60.3 | 7.0 | 9.0 | 4 |
| 383 | 4631h14 | 32.6 | 7.0 | 9.0 | 8 |
| 384 | 4652h45 | 52.3 | 7.0 | 9.0 | 4 |
| 385 | 4681h53 | 52.9 | 7.0 | 9.0 | 4 |
| 386 | 4686h35 | 51.0 | 7.0 | 9.0 | 4 |
| 387 | 771p46-18-1 | 41.2 | 7.0 | 9.0 | 8 |
| 539 | 4519h24 | 46.9 | 7.0 | 9.0 | 7 |
| 540 | 4528h1 | 55.1 | 7.0 | 9.0 | 7 |
| 541 | 4529h25 | 61.5 | 7.0 | 9.0 | 7 |
| 542 | 4557h38 | 51.7 | 7.0 | 9.0 | 7 |
| 543 | 4626h23 | 63.5 | 7.0 | 9.0 | 8 |
| 544 | 4639h102 | 37.9 | 7.0 | 9.0 | 7 |
| 545 | 4643h29 | 24.2 | 7.0 | 9.0 | 7 |
| 546 | 4646h1 | 44.7 | 9.0 | 9.0 | 7 |
| 547 | 4675h11 | 43.6 | 7.0 | 9.0 | 7 |
| 548 | 4518h20 | 47.7 |  |  | 6 |
| 549 | 3902h3-18-3 | 67.0 | 7.0 | 9.0 | 7 |
| 550 | 4822h19 | 31.6 |  |  | 4 |
| 551 | 3408h218-18-173 | 49.4 | 5.0 | 9.0 | 11 |
| 552 | 3683h46-18-59 | 60.5 | 8.0 | 9.0 | 7 |
| 554 | 4428h48-19-11 | 36.5 |  |  | 4 |
| 555 | 4778h29 | 52.4 |  |  | 5 |
| 556 | 4779h57 | 35.8 |  |  | 5 |
| 557 | 4812h7 | 44.6 |  |  | 5 |
| 560 | 4626h10-20-24 | 30.5 | 3.0 |  | 2 |

Table S3. Individual F_2_ data (8 informative families): plant, family, status, *Fr-A2* genotypes.

Selective genotyping: the block genotyped before freezing (152 plants) and the block of survivors (52 plants). Individual plant identity could not be reliably matched between the blocks, and every analysis in this work conditions on family membership alone.

| **Plant (F_2_)** | **Family** | **Status** | **S1077313** | **S2269949** |
| --- | --- | --- | --- | --- |
| 3320-3-5 | 3320-3 | survivor | HEX | Heterozygote |
| 3320-3-1 | 3320-3 | before selection | FAM | Heterozygote |
| 3320-3-10 | 3320-3 | before selection | HEX | Heterozygote |
| 3320-3-11 | 3320-3 | before selection | FAM | HEX |
| 3320-3-12 | 3320-3 | before selection | HEX | Heterozygote |
| 3320-3-13 | 3320-3 | before selection | FAM | HEX |
| 3320-3-14 | 3320-3 | before selection | FAM | HEX |
| 3320-3-15 | 3320-3 | before selection | HEX | Heterozygote |
| 3320-3-16 | 3320-3 | before selection | Heterozygote | HEX |
| 3320-3-17 | 3320-3 | before selection | FAM | Heterozygote |
| 3320-3-2 | 3320-3 | before selection | FAM | HEX |
| 3320-3-3 | 3320-3 | before selection | FAM | HEX |
| 3320-3-4 | 3320-3 | before selection | HEX | FAM |
| 3320-3-5 | 3320-3 | before selection | HEX | Heterozygote |
| 3320-3-6 | 3320-3 | before selection | HEX | Heterozygote |
| 3320-3-7 | 3320-3 | before selection | FAM | HEX |
| 3320-3-8 | 3320-3 | before selection | FAM | HEX |
| 3320-3-9 | 3320-3 | before selection | HEX | Heterozygote |
| 3321-3-1 | 3321-3 | survivor | HEX | Heterozygote |
| 3321-3-10 | 3321-3 | survivor | HEX | FAM |
| 3321-3-11 | 3321-3 | survivor | HEX | FAM |
| 3321-3-17 | 3321-3 | survivor | HEX | FAM |
| 3321-3-19 | 3321-3 | survivor | HEX | FAM |
| 3321-3-2 | 3321-3 | survivor | HEX | FAM |
| 3321-3-3 | 3321-3 | survivor | HEX | Heterozygote |
| 3321-3-4 | 3321-3 | survivor | HEX | FAM |
| 3321-3-1 | 3321-3 | before selection | HEX | Heterozygote |
| 3321-3-10 | 3321-3 | before selection | HEX | HEX |
| 3321-3-11 | 3321-3 | before selection | HEX | FAM |
| 3321-3-12 | 3321-3 | before selection | HEX | FAM |
| 3321-3-13 | 3321-3 | before selection | HEX | FAM |
| 3321-3-14 | 3321-3 | before selection | HEX | FAM |
| 3321-3-15 | 3321-3 | before selection | HEX | FAM |
| 3321-3-16 | 3321-3 | before selection | HEX | FAM |
| 3321-3-17 | 3321-3 | before selection | HEX | FAM |
| 3321-3-18 | 3321-3 | before selection | HEX | FAM |
| 3321-3-19 | 3321-3 | before selection | HEX | FAM |
| 3321-3-2 | 3321-3 | before selection | HEX | FAM |
| 3321-3-20 | 3321-3 | before selection | HEX | Heterozygote |
| 3321-3-21 | 3321-3 | before selection | HEX | FAM |
| 3321-3-22 | 3321-3 | before selection | HEX | Heterozygote |
| 3321-3-3 | 3321-3 | before selection | HEX | Heterozygote |
| 3321-3-4 | 3321-3 | before selection | HEX | FAM |
| 3321-3-5 | 3321-3 | before selection | HEX | FAM |
| 3321-3-6 | 3321-3 | before selection | HEX | FAM |
| 3321-3-7 | 3321-3 | before selection | HEX | FAM |
| 3321-3-8 | 3321-3 | before selection | HEX | FAM |
| 3321-3-9 | 3321-3 | before selection | HEX | FAM |
| 3321-5-1 | 3321-5 | survivor | HEX | FAM |
| 3321-5-14 | 3321-5 | survivor | HEX | FAM |
| 3321-5-15 | 3321-5 | survivor | HEX | FAM |
| 3321-5-19 | 3321-5 | survivor | HEX | FAM |
| 3321-5-20 | 3321-5 | survivor | HEX | FAM |
| 3321-5-4 | 3321-5 | survivor | HEX | FAM |
| 3321-5-5 | 3321-5 | survivor | HEX | FAM |
| 3321-5-6 | 3321-5 | survivor | HEX | FAM |
| 3321-5-1 | 3321-5 | before selection | HEX | FAM |
| 3321-5-10 | 3321-5 | before selection | HEX | FAM |
| 3321-5-11 | 3321-5 | before selection | HEX | FAM |
| 3321-5-12 | 3321-5 | before selection | HEX | FAM |
| 3321-5-13 | 3321-5 | before selection | HEX | FAM |
| 3321-5-14 | 3321-5 | before selection | HEX | FAM |
| 3321-5-15 | 3321-5 | before selection | No Call | FAM |
| 3321-5-16 | 3321-5 | before selection | HEX | FAM |
| 3321-5-17 | 3321-5 | before selection | HEX | FAM |
| 3321-5-18 | 3321-5 | before selection | HEX | FAM |
| 3321-5-19 | 3321-5 | before selection | HEX | FAM |
| 3321-5-2 | 3321-5 | before selection | HEX | FAM |
| 3321-5-3 | 3321-5 | before selection | HEX | FAM |
| 3321-5-4 | 3321-5 | before selection | Undetermined | Undetermined |
| 3321-5-5 | 3321-5 | before selection | HEX | FAM |
| 3321-5-6 | 3321-5 | before selection | HEX | FAM |
| 3321-5-7 | 3321-5 | before selection | HEX | FAM |
| 3321-5-8 | 3321-5 | before selection | HEX | HEX |
| 3321-5-9 | 3321-5 | before selection | HEX | HEX |
| 3321-6-1 | 3321-6 | survivor | HEX | FAM |
| 3321-6-11 | 3321-6 | survivor | HEX | Heterozygote |
| 3321-6-16 | 3321-6 | survivor | HEX | FAM |
| 3321-6-17 | 3321-6 | survivor | HEX | FAM |
| 3321-6-18 | 3321-6 | survivor | HEX | FAM |
| 3321-6-7 | 3321-6 | survivor | HEX | FAM |
| 3321-6-1 | 3321-6 | before selection | HEX | FAM |
| 3321-6-10 | 3321-6 | before selection | FAM | HEX |
| 3321-6-11 | 3321-6 | before selection | HEX | Heterozygote |
| 3321-6-12 | 3321-6 | before selection | HEX | Heterozygote |
| 3321-6-13 | 3321-6 | before selection | HEX | Undetermined |
| 3321-6-14 | 3321-6 | before selection | FAM | HEX |
| 3321-6-15 | 3321-6 | before selection | HEX | FAM |
| 3321-6-16 | 3321-6 | before selection | HEX | FAM |
| 3321-6-17 | 3321-6 | before selection | HEX | FAM |
| 3321-6-18 | 3321-6 | before selection | HEX | FAM |
| 3321-6-19 | 3321-6 | before selection | HEX | FAM |
| 3321-6-2 | 3321-6 | before selection | HEX | FAM |
| 3321-6-3 | 3321-6 | before selection | HEX | FAM |
| 3321-6-4 | 3321-6 | before selection | FAM | HEX |
| 3321-6-5 | 3321-6 | before selection | FAM | HEX |
| 3321-6-6 | 3321-6 | before selection | HEX | Heterozygote |
| 3321-6-7 | 3321-6 | before selection | HEX | FAM |
| 3321-6-8 | 3321-6 | before selection | HEX | Heterozygote |
| 3321-6-9 | 3321-6 | before selection | HEX | FAM |
| 3321-7-1 | 3321-7 | survivor | HEX | FAM |
| 3321-7-14 | 3321-7 | survivor | HEX | FAM |
| 3321-7-17 | 3321-7 | survivor | HEX | FAM |
| 3321-7-5 | 3321-7 | survivor | HEX | FAM |
| 3321-7-6 | 3321-7 | survivor | HEX | FAM |
| 3321-7-1 | 3321-7 | before selection | HEX | FAM |
| 3321-7-10 | 3321-7 | before selection | HEX | FAM |
| 3321-7-11 | 3321-7 | before selection | HEX | FAM |
| 3321-7-12 | 3321-7 | before selection | HEX | FAM |
| 3321-7-13 | 3321-7 | before selection | HEX | FAM |
| 3321-7-14 | 3321-7 | before selection | HEX | Heterozygote |
| 3321-7-15 | 3321-7 | before selection | HEX | FAM |
| 3321-7-16 | 3321-7 | before selection | HEX | FAM |
| 3321-7-17 | 3321-7 | before selection | HEX | FAM |
| 3321-7-18 | 3321-7 | before selection | HEX | FAM |
| 3321-7-2 | 3321-7 | before selection | HEX | HEX |
| 3321-7-3 | 3321-7 | before selection | HEX | Heterozygote |
| 3321-7-4 | 3321-7 | before selection | HEX | FAM |
| 3321-7-5 | 3321-7 | before selection | HEX | FAM |
| 3321-7-6 | 3321-7 | before selection | HEX | FAM |
| 3321-7-7 | 3321-7 | before selection | HEX | Heterozygote |
| 3321-7-8 | 3321-7 | before selection | HEX | FAM |
| 3321-7-9 | 3321-7 | before selection | HEX | FAM |
| 3324-7-3 | 3324-7 | survivor | Undetermined | Heterozygote |
| 3324-7-9 | 3324-7 | survivor | HEX | FAM |
| 3324-7-1 | 3324-7 | before selection | FAM | Heterozygote |
| 3324-7-10 | 3324-7 | before selection | HEX | FAM |
| 3324-7-11 | 3324-7 | before selection | HEX | FAM |
| 3324-7-12 | 3324-7 | before selection | HEX | FAM |
| 3324-7-13 | 3324-7 | before selection | HEX | FAM |
| 3324-7-14 | 3324-7 | before selection | HEX | Undetermined |
| 3324-7-15 | 3324-7 | before selection | HEX | FAM |
| 3324-7-16 | 3324-7 | before selection | HEX | FAM |
| 3324-7-17 | 3324-7 | before selection | HEX | FAM |
| 3324-7-2 | 3324-7 | before selection | HEX | FAM |
| 3324-7-3 | 3324-7 | before selection | HEX | Heterozygote |
| 3324-7-4 | 3324-7 | before selection | HEX | Heterozygote |
| 3324-7-5 | 3324-7 | before selection | Undetermined | FAM |
| 3324-7-6 | 3324-7 | before selection | HEX | FAM |
| 3324-7-7 | 3324-7 | before selection | HEX | FAM |
| 3324-7-8 | 3324-7 | before selection | HEX | FAM |
| 3324-7-9 | 3324-7 | before selection | HEX | FAM |
| 3325-3-1 | 3325-3 | survivor | Undetermined | HEX |
| 3325-3-11 | 3325-3 | survivor | HEX | FAM |
| 3325-3-12 | 3325-3 | survivor | HEX | FAM |
| 3325-3-16 | 3325-3 | survivor | HEX | FAM |
| 3325-3-18 | 3325-3 | survivor | HEX | FAM |
| 3325-3-19 | 3325-3 | survivor | HEX | FAM |
| 3325-3-20 | 3325-3 | survivor | HEX | FAM |
| 3325-3-4 | 3325-3 | survivor | Undetermined | FAM |
| 3325-3-1 | 3325-3 | before selection | FAM | HEX |
| 3325-3-10 | 3325-3 | before selection | HEX | FAM |
| 3325-3-11 | 3325-3 | before selection | HEX | FAM |
| 3325-3-12 | 3325-3 | before selection | HEX | FAM |
| 3325-3-13 | 3325-3 | before selection | HEX | Heterozygote |
| 3325-3-14 | 3325-3 | before selection | HEX | FAM |
| 3325-3-15 | 3325-3 | before selection | HEX | FAM |
| 3325-3-16 | 3325-3 | before selection | HEX | FAM |
| 3325-3-17 | 3325-3 | before selection | HEX | FAM |
| 3325-3-18 | 3325-3 | before selection | HEX | Heterozygote |
| 3325-3-19 | 3325-3 | before selection | HEX | FAM |
| 3325-3-2 | 3325-3 | before selection | HEX | FAM |
| 3325-3-20 | 3325-3 | before selection | HEX | FAM |
| 3325-3-3 | 3325-3 | before selection | HEX | FAM |
| 3325-3-4 | 3325-3 | before selection | HEX | FAM |
| 3325-3-5 | 3325-3 | before selection | HEX | FAM |
| 3325-3-6 | 3325-3 | before selection | HEX | Heterozygote |
| 3325-3-7 | 3325-3 | before selection | HEX | FAM |
| 3325-3-8 | 3325-3 | before selection | HEX | Heterozygote |
| 3325-3-9 | 3325-3 | before selection | FAM | HEX |
| 3325-5-1 | 3325-5 | survivor | HEX | FAM |
| 3325-5-10 | 3325-5 | survivor | HEX | FAM |
| 3325-5-11 | 3325-5 | survivor | No Call | FAM |
| 3325-5-12 | 3325-5 | survivor | FAM | Heterozygote |
| 3325-5-16 | 3325-5 | survivor | HEX | FAM |
| 3325-5-17 | 3325-5 | survivor | HEX | FAM |
| 3325-5-18 | 3325-5 | survivor | HEX | FAM |
| 3325-5-2 | 3325-5 | survivor | HEX | FAM |
| 3325-5-3 | 3325-5 | survivor | HEX | FAM |
| 3325-5-4 | 3325-5 | survivor | HEX | HEX |
| 3325-5-6 | 3325-5 | survivor | HEX | FAM |
| 3325-5-7 | 3325-5 | survivor | HEX | Heterozygote |
| 3325-5-8 | 3325-5 | survivor | HEX | Heterozygote |
| 3325-5-9 | 3325-5 | survivor | HEX | Heterozygote |
| 3325-5-1 | 3325-5 | before selection | HEX | FAM |
| 3325-5-10 | 3325-5 | before selection | HEX | FAM |
| 3325-5-11 | 3325-5 | before selection | HEX | FAM |
| 3325-5-12 | 3325-5 | before selection | HEX | Heterozygote |
| 3325-5-13 | 3325-5 | before selection | FAM | Heterozygote |
| 3325-5-14 | 3325-5 | before selection | HEX | FAM |
| 3325-5-15 | 3325-5 | before selection | FAM | HEX |
| 3325-5-16 | 3325-5 | before selection | HEX | Heterozygote |
| 3325-5-17 | 3325-5 | before selection | HEX | FAM |
| 3325-5-18 | 3325-5 | before selection | HEX | Heterozygote |
| 3325-5-19 | 3325-5 | before selection | HEX | Heterozygote |
| 3325-5-2 | 3325-5 | before selection | HEX | FAM |
| 3325-5-20 | 3325-5 | before selection | HEX | Heterozygote |
| 3325-5-3 | 3325-5 | before selection | HEX | Heterozygote |
| 3325-5-4 | 3325-5 | before selection | HEX | FAM |
| 3325-5-5 | 3325-5 | before selection | HEX | FAM |
| 3325-5-6 | 3325-5 | before selection | HEX | Heterozygote |
| 3325-5-7 | 3325-5 | before selection | HEX | Heterozygote |
| 3325-5-8 | 3325-5 | before selection | HEX | FAM |
| 3325-5-9 | 3325-5 | before selection | HEX | FAM |

## Supplementary Figures S1 to S4: photographs of the controlled freezing method

Figure S1. Visual scoring of regrowth after freezing on a 0 to 9 scale, 7 to 9 days after freezing, where 0 denotes complete absence of regrowth and 9 denotes maximum regrowth.

Figure S2. Differential plant survival in a box after freezing.

Figure S3. Concrete racks at the experimental station of the P. P. Lukyanenko National Grain Center for assessing winter and frost hardiness.

Figure S4. Visual scoring of overwintering under field conditions on the 0 to 9 scale, calibrated each year against the standards Krupinka and Donchanka.

Table S4. Origin of the studied accessions: originator, country, year of entry into State Variety Trials and origin group used in the adjusted models (Table S8). Year of entry is given as reported by the originator; for breeding lines it is the year the line was entered. Origin groups: NCZ Krasnodar, P. P. Lukyanenko National Grain Center; ANC Donskoy, Agricultural Research Center Donskoy; other Russian; foreign.

| **sample_id** | **Cultivar / line** | **Originator** | **Country** | **Year of entry into State Variety Trials** | **Origin group** |
| --- | --- | --- | --- | --- | --- |
| 20 | Krupinka | P. P. Lukyanenko National Grain Center, Russia | Russia | 2005 | NCZ Krasnodar |
| 40 | Krucha | P. P. Lukyanenko National Grain Center, Russia | Russia | 2015 | NCZ Krasnodar |
| 203 | Kristall 2 | P. P. Lukyanenko National Grain Center, Russia | Russia | 1983 | NCZ Krasnodar |
| 204 | Leukurum 21 | P. P. Lukyanenko National Grain Center, Russia | Russia | 1996 | NCZ Krasnodar |
| 205 | Alena | P. P. Lukyanenko National Grain Center, Russia | Russia | 2000 | NCZ Krasnodar |
| 206 | Kermen | P. P. Lukyanenko National Grain Center, Russia | Russia | 2006 | NCZ Krasnodar |
| 207 | Uniya | P. P. Lukyanenko National Grain Center, Russia | Russia | 2008 | NCZ Krasnodar |
| 208 | Zolotko | P. P. Lukyanenko National Grain Center, Russia | Russia | 2009 | NCZ Krasnodar |
| 209 | Laska | P. P. Lukyanenko National Grain Center, Russia | Russia | 2011 | NCZ Krasnodar |
| 210 | Odari | P. P. Lukyanenko National Grain Center, Russia | Russia | 2017 | NCZ Krasnodar |
| 211 | Altana | P. P. Lukyanenko National Grain Center, Russia | Russia | 2014 | NCZ Krasnodar |
| 212 | Kordon | P. P. Lukyanenko National Grain Center, Russia | Russia | 2018 | NCZ Krasnodar |
| 213 | Dobryana | P. P. Lukyanenko National Grain Center, Russia | Russia | 2016 | NCZ Krasnodar |
| 214 | Sinora | P. P. Lukyanenko National Grain Center, Russia | Russia | 2022 | NCZ Krasnodar |
| 215 | Donchanka | Agricultural Research Center Donskoy, Russia | Russia | 2001 | ANC Donskoy |
| 216 | Kurant | Agricultural Research Center Donskoy, Russia | Russia | 2001 | ANC Donskoy |
| 217 | Kristella | Agricultural Research Center Donskoy, Russia | Russia | 2008 | ANC Donskoy |
| 218 | Amazonka | Agricultural Research Center Donskoy, Russia | Russia | 2009 | ANC Donskoy |
| 219 | Agat donskoy | Agricultural Research Center Donskoy, Russia | Russia | 2012 | ANC Donskoy |
| 220 | Lazurit | Agricultural Research Center Donskoy, Russia | Russia | 2014 | ANC Donskoy |
| 221 | Oniks | Agricultural Research Center Donskoy, Russia | Russia | 2015 | ANC Donskoy |
| 222 | Diona | Agricultural Research Center Donskoy, Russia | Russia | 2016 | ANC Donskoy |
| 223 | Kiprida | Agricultural Research Center Donskoy, Russia | Russia | 2018 | ANC Donskoy |
| 224 | Teyya | Agricultural Research Center Donskoy, Russia | Russia | 2015 | ANC Donskoy |
| 225 | Eyrena | Agricultural Research Center Donskoy, Russia | Russia | 2017 | ANC Donskoy |
| 226 | Yakhont (760/10) | Agricultural Research Center Donskoy, Russia | Russia | 2018 | ANC Donskoy |
| 227 | Yubilyarka (330/10) | Agricultural Research Center Donskoy, Russia | Russia | 2019 | ANC Donskoy |
| 228 | Yantarina | Agricultural Research Center Donskoy, Russia | Russia | 2020 | ANC Donskoy |
| 229 | Uslada | Agricultural Research Center Donskoy, Russia | Russia | 2021 | ANC Donskoy |
| 230 | Zoloto Dona (993/ 12) | Agricultural Research Center Donskoy, Russia | Russia | 2021 | ANC Donskoy |
| 231 | 796/11 | Agricultural Research Center Donskoy, Russia | Russia | 2010 | ANC Donskoy |
| 232 | 589/13 | Agricultural Research Center Donskoy, Russia | Russia | 2005 | ANC Donskoy |
| 233 | 737/11 | Agricultural Research Center Donskoy, Russia | Russia | 2008 | ANC Donskoy |
| 234 | 655/13 (Solntsedar) | Agricultural Research Center Donskoy, Russia | Russia | 2013 | ANC Donskoy |
| 235 | 533/14 | Agricultural Research Center Donskoy, Russia | Russia | 2005 | ANC Donskoy |
| 236 | Prikumchanka | Prikumskaya Experimental Breeding Station, Russia | Russia | 1995 | other Russian |
| 237 | Stepnoy yantar | Prikumskaya Experimental Breeding Station, Russia | Russia | 2014 | other Russian |
| 238 | Zhivitsa | Agricultural Research Center Donskoy, Russia | Russia | 2011 | ANC Donskoy |
| 239 | Bagryanitsa (Italikum) | Prikumskaya Experimental Breeding Station, Russia | Russia | 2010 | other Russian |
| 240 | Perlina odesskaya | Plant Breeding and Genetics Institute, National Center of Seed and Cultivar Investigation, Odesa, Ukraine | Ukraine | 2006 | foreign |
| 241 | Burshtyn | Plant Breeding and Genetics Institute, National Center of Seed and Cultivar Investigation, Odesa, Ukraine | Ukraine | 2007 | foreign |
| 242 | Kontinent | Plant Breeding and Genetics Institute, National Center of Seed and Cultivar Investigation, Odesa, Ukraine | Ukraine | 2015 | foreign |
| 243 | Akveduk | Plant Breeding and Genetics Institute, National Center of Seed and Cultivar Investigation, Odesa, Ukraine | Ukraine | 2014 | foreign |
| 244 | Andromeda | Institute of Irrigated Agriculture, NAAS (Kherson) and Plant Breeding and Genetics Institute, Odesa, Ukraine | Ukraine | 2013 | foreign |
| 245 | Dnepryana | Institute of Irrigated Agriculture, NAAS (Kherson) and Plant Breeding and Genetics Institute, Odesa, Ukraine | Ukraine | 2000 | foreign |
| 246 | Kharkovskaya 32 | V. Ya. Yuriev Institute of Plant Production, NAAS, Ukraine | Ukraine | 1996 | foreign |
| 247 | Leukurum 36 | V. Ya. Yuriev Institute of Plant Production, NAAS, Ukraine | Ukraine | 1986 | foreign |
| 248 | Afina | V. Ya. Yuriev Institute of Plant Production, NAAS, Ukraine | Ukraine | 2013 | foreign |
| 249 | Makar | V. Ya. Yuriev Institute of Plant Production, NAAS, Ukraine | Ukraine | 2014 | foreign |
| 250 | Belgorodskaya yantarnaya | V. Ya. Yuriev Institute of Plant Production, Ukraine and Sativa Ltd, Russia | Russia/Ukraine | 2015 | foreign |
| 251 | Temnyy yantar | Sativa Ltd, Russia | Russia | 2024 | other Russian |
| 252 | Koshelevskaya | Koshelevsky Posad Ltd, Russia | Russia | 2022 | other Russian |
| 253 | Shulyndinka | V. Ya. Yuriev Institute of Plant Production, NAAS, Ukraine | Ukraine | 2013 | foreign |
| 254 | Pributkova | V. Ya. Yuriev Institute of Plant Production, NAAS, Ukraine | Ukraine | 2019 | foreign |
| 255 | Yagut | Research Institute of Crop Husbandry, Azerbaijan | Azerbaijan | 1998 | foreign |
| 256 | DF -104/85 | INCDA Fundulea, Romania | Romania | 1985 | foreign |
| 257 | Pandur | Probstdorfer Saatzucht, Austria | Austria | 1996 | foreign |
| 258 | Elidur | RAGT Semences, France | France | 2003 | foreign |
| 259 | Rodur | RAGT Semences, France | France | 1984 | foreign |
| 260 | Condur | RAGT Semences, France | France | 1973 | foreign |
| 261 | Blindur/DF 38-86/3 | INCDA Fundulea, Romania | Romania | 1986 | foreign |
| 262 | Rumyniya DF-01058-1-101 | INCDA Fundulea, Romania | Romania | 2001 | foreign |
| 263 | Rumyniya DF-00091-14-002 | INCDA Fundulea, Romania | Romania | 2014 | foreign |
| 264 | Martondur 1 | Agricultural Research Institute of the Hungarian Academy of Sciences, Hungary | Hungary | 1996 | foreign |
| 265 | MVTD 12-97 | Agricultural Research Institute of the Hungarian Academy of Sciences, Hungary | Hungary | 1997 | foreign |
| 266 | MVTD 12-99 | Agricultural Research Institute of the Hungarian Academy of Sciences, Hungary | Hungary | 1999 | foreign |
| 267 | MVTD 15-98 | Agricultural Research Institute of the Hungarian Academy of Sciences, Hungary | Hungary | 1998 | foreign |
| 268 | MV Makaroni | Agricultural Research Institute of the Hungarian Academy of Sciences, Hungary | Hungary | 2001 | foreign |
| 269 | MV HUNDUR | Agricultural Research Institute of the Hungarian Academy of Sciences, Hungary | Hungary | 2008 | foreign |
| 270 | MV PENNEDUR | Agricultural Research Institute of the Hungarian Academy of Sciences, Hungary | Hungary | 2007 | foreign |
| 271 | Wintergold | Südwestdeutsche Saatzucht, Germany | Germany | 2011 | foreign |
| 272 | AMA 11855t85-90 | Dagestan Experimental Station, branch of VIR, Russia | Russia | 2018 | other Russian |
| 273 | AMA 15908ds1122-07 | Dagestan Experimental Station, branch of VIR, Russia | Russia | 2007 | other Russian |
| 274 | 21545t | Dagestan Experimental Station, branch of VIR, Russia | Russia | 2019 | other Russian |
| 275 | 3561h28 | P. P. Lukyanenko National Grain Center, Russia | Russia | 2012 | NCZ Krasnodar |
| 276 | 3622h46 | P. P. Lukyanenko National Grain Center, Russia | Russia | 2013 | NCZ Krasnodar |
| 277 | 3591h96 | P. P. Lukyanenko National Grain Center, Russia | Russia | 2012 | NCZ Krasnodar |
| 278 | 3336h6-13-162 | P. P. Lukyanenko National Grain Center, Russia | Russia | 2011 | NCZ Krasnodar |
| 279 | 3680h62 (Belka) | P. P. Lukyanenko National Grain Center, Russia | Russia | 2025 | NCZ Krasnodar |
| 280 | 3796h33 | P. P. Lukyanenko National Grain Center, Russia | Russia | 2013 | NCZ Krasnodar |
| 326 | 3850h69 (Tsel) | P. P. Lukyanenko National Grain Center, Russia | Russia | 2024 | NCZ Krasnodar |
| 327 | 3527h4-14-3 | P. P. Lukyanenko National Grain Center, Russia | Russia | 2012 | NCZ Krasnodar |
| 328 | 3609h36-14-4 (Bella) | P. P. Lukyanenko National Grain Center, Russia | Russia | 2024 | NCZ Krasnodar |
| 329 | 3982h42 | P. P. Lukyanenko National Grain Center, Russia | Russia | 2014 | NCZ Krasnodar |
| 330 | 3359h18-15-174 | P. P. Lukyanenko National Grain Center, Russia | Russia | 2011 | NCZ Krasnodar |
| 331 | 3896h145 | P. P. Lukyanenko National Grain Center, Russia | Russia | 2013 | NCZ Krasnodar |
| 332 | 3934h148 | P. P. Lukyanenko National Grain Center, Russia | Russia | 2014 | NCZ Krasnodar |
| 333 | 3934h155 | P. P. Lukyanenko National Grain Center, Russia | Russia | 2014 | NCZ Krasnodar |
| 334 | 4089h94 | P. P. Lukyanenko National Grain Center, Russia | Russia | 2014 | NCZ Krasnodar |
| 335 | 4135h7 | P. P. Lukyanenko National Grain Center, Russia | Russia | 2014 | NCZ Krasnodar |
| 336 | 4218h77 | P. P. Lukyanenko National Grain Center, Russia | Russia | 2014 | NCZ Krasnodar |
| 337 | 4169h75 | P. P. Lukyanenko National Grain Center, Russia | Russia | 2014 | NCZ Krasnodar |
| 338 | 4192h94 | P. P. Lukyanenko National Grain Center, Russia | Russia | 2014 | NCZ Krasnodar |
| 339 | 4192h98 | P. P. Lukyanenko National Grain Center, Russia | Russia | 2014 | NCZ Krasnodar |
| 340 | 4336h7 (Zashchita) | P. P. Lukyanenko National Grain Center, Russia | Russia | 2026 | NCZ Krasnodar |
| 341 | 4336h24 | P. P. Lukyanenko National Grain Center, Russia | Russia | 2014 | NCZ Krasnodar |
| 342 | 4337h3 | P. P. Lukyanenko National Grain Center, Russia | Russia | 2014 | NCZ Krasnodar |
| 343 | 4341h1 | P. P. Lukyanenko National Grain Center, Russia | Russia | 2014 | NCZ Krasnodar |
| 344 | 4342h23 | P. P. Lukyanenko National Grain Center, Russia | Russia | 2014 | NCZ Krasnodar |
| 345 | 3596h56-15-22 (Dara) | P. P. Lukyanenko National Grain Center, Russia | Russia | 2021 | NCZ Krasnodar |
| 346 | 3596h56-15-69 | P. P. Lukyanenko National Grain Center, Russia | Russia | 2012 | NCZ Krasnodar |
| 347 | 4306h15 | P. P. Lukyanenko National Grain Center, Russia | Russia | 2014 | NCZ Krasnodar |
| 348 | 4320h38 | P. P. Lukyanenko National Grain Center, Russia | Russia | 2014 | NCZ Krasnodar |
| 349 | 4341h68 | P. P. Lukyanenko National Grain Center, Russia | Russia | 2014 | NCZ Krasnodar |
| 350 | 4353h47 | P. P. Lukyanenko National Grain Center, Russia | Russia | 2014 | NCZ Krasnodar |
| 351 | 4383h72 | P. P. Lukyanenko National Grain Center, Russia | Russia | 2014 | NCZ Krasnodar |
| 352 | 4387h148 | P. P. Lukyanenko National Grain Center, Russia | Russia | 2014 | NCZ Krasnodar |
| 353 | 4392h17 | P. P. Lukyanenko National Grain Center, Russia | Russia | 2014 | NCZ Krasnodar |
| 354 | 4416h49 skorosp. | P. P. Lukyanenko National Grain Center, Russia | Russia | 2014 | NCZ Krasnodar |
| 355 | 4419h45 | P. P. Lukyanenko National Grain Center, Russia | Russia | 2015 | NCZ Krasnodar |
| 356 | 4424h4 | P. P. Lukyanenko National Grain Center, Russia | Russia | 2015 | NCZ Krasnodar |
| 357 | 4424h96 | P. P. Lukyanenko National Grain Center, Russia | Russia | 2015 | NCZ Krasnodar |
| 358 | 4428h110 | P. P. Lukyanenko National Grain Center, Russia | Russia | 2015 | NCZ Krasnodar |
| 359 | 4430h50 | P. P. Lukyanenko National Grain Center, Russia | Russia | 2015 | NCZ Krasnodar |
| 360 | 4432h24 | P. P. Lukyanenko National Grain Center, Russia | Russia | 2015 | NCZ Krasnodar |
| 361 | 4432h45 | P. P. Lukyanenko National Grain Center, Russia | Russia | 2015 | NCZ Krasnodar |
| 362 | 4433h52 | P. P. Lukyanenko National Grain Center, Russia | Russia | 2015 | NCZ Krasnodar |
| 363 | 4433h90 | P. P. Lukyanenko National Grain Center, Russia | Russia | 2015 | NCZ Krasnodar |
| 364 | 4466h183 | P. P. Lukyanenko National Grain Center, Russia | Russia | 2015 | NCZ Krasnodar |
| 365 | 4467h18 | P. P. Lukyanenko National Grain Center, Russia | Russia | 2015 | NCZ Krasnodar |
| 366 | 2468h10-06M-17-10 | P. P. Lukyanenko National Grain Center, Russia | Russia | 2007 | NCZ Krasnodar |
| 367 | 3336h43-17-128 | P. P. Lukyanenko National Grain Center, Russia | Russia | 2011 | NCZ Krasnodar |
| 368 | 3903h9-17-3 | P. P. Lukyanenko National Grain Center, Russia | Russia | 2014 | NCZ Krasnodar |
| 369 | 4167h48-17-12 | P. P. Lukyanenko National Grain Center, Russia | Russia | 2014 | NCZ Krasnodar |
| 370 | 4250hK-19 | P. P. Lukyanenko National Grain Center, Russia | Russia | 2014 | NCZ Krasnodar |
| 371 | 3902h3-18-31 | P. P. Lukyanenko National Grain Center, Russia | Russia | 2014 | NCZ Krasnodar |
| 372 | 3914h38-18-8 | P. P. Lukyanenko National Grain Center, Russia | Russia | 2014 | NCZ Krasnodar |
| 373 | 4477h2 | P. P. Lukyanenko National Grain Center, Russia | Russia | 2015 | NCZ Krasnodar |
| 374 | 4482h8 | P. P. Lukyanenko National Grain Center, Russia | Russia | 2015 | NCZ Krasnodar |
| 375 | 4505h38 | P. P. Lukyanenko National Grain Center, Russia | Russia | 2016 | NCZ Krasnodar |
| 376 | 4509h13 | P. P. Lukyanenko National Grain Center, Russia | Russia | 2016 | NCZ Krasnodar |
| 377 | 4517h25 | P. P. Lukyanenko National Grain Center, Russia | Russia | 2016 | NCZ Krasnodar |
| 378 | 4521h13 | P. P. Lukyanenko National Grain Center, Russia | Russia | 2016 | NCZ Krasnodar |
| 379 | 4574h8 | P. P. Lukyanenko National Grain Center, Russia | Russia | 2016 | NCZ Krasnodar |
| 380 | 4619h9 | P. P. Lukyanenko National Grain Center, Russia | Russia | 2017 | NCZ Krasnodar |
| 381 | 4619h34 | P. P. Lukyanenko National Grain Center, Russia | Russia | 2017 | NCZ Krasnodar |
| 382 | 4626h15 | P. P. Lukyanenko National Grain Center, Russia | Russia | 2017 | NCZ Krasnodar |
| 383 | 4631h14 | P. P. Lukyanenko National Grain Center, Russia | Russia | 2017 | NCZ Krasnodar |
| 384 | 4652h45 | P. P. Lukyanenko National Grain Center, Russia | Russia | 2017 | NCZ Krasnodar |
| 385 | 4681h53 | P. P. Lukyanenko National Grain Center, Russia | Russia | 2017 | NCZ Krasnodar |
| 386 | 4686h35 | P. P. Lukyanenko National Grain Center, Russia | Russia | 2017 | NCZ Krasnodar |
| 387 | 771p46-18-1 | P. P. Lukyanenko National Grain Center, Russia | Russia | 2010 | NCZ Krasnodar |
| 539 | 4519h24 | P. P. Lukyanenko National Grain Center, Russia | Russia | 2016 | NCZ Krasnodar |
| 540 | 4528h1 | P. P. Lukyanenko National Grain Center, Russia | Russia | 2016 | NCZ Krasnodar |
| 541 | 4529h25 | P. P. Lukyanenko National Grain Center, Russia | Russia | 2016 | NCZ Krasnodar |
| 542 | 4557h38 | P. P. Lukyanenko National Grain Center, Russia | Russia | 2016 | NCZ Krasnodar |
| 543 | 4626h23 | P. P. Lukyanenko National Grain Center, Russia | Russia | 2017 | NCZ Krasnodar |
| 544 | 4639h102 | P. P. Lukyanenko National Grain Center, Russia | Russia | 2017 | NCZ Krasnodar |
| 545 | 4643h29 | P. P. Lukyanenko National Grain Center, Russia | Russia | 2017 | NCZ Krasnodar |
| 546 | 4646h1 | P. P. Lukyanenko National Grain Center, Russia | Russia | 2017 | NCZ Krasnodar |
| 547 | 4675h11 | P. P. Lukyanenko National Grain Center, Russia | Russia | 2017 | NCZ Krasnodar |
| 548 | 4518h20 | P. P. Lukyanenko National Grain Center, Russia | Russia | 2016 | NCZ Krasnodar |
| 549 | 3902h3-18-3 | P. P. Lukyanenko National Grain Center, Russia | Russia | 2014 | NCZ Krasnodar |
| 550 | 4822h19 | P. P. Lukyanenko National Grain Center, Russia | Russia | 2018 | NCZ Krasnodar |
| 551 | 3408h218-18-173 | P. P. Lukyanenko National Grain Center, Russia | Russia | 2011 | NCZ Krasnodar |
| 552 | 3683h46-18-59 | P. P. Lukyanenko National Grain Center, Russia | Russia | 2013 | NCZ Krasnodar |
| 554 | 4428h48-19-11 | P. P. Lukyanenko National Grain Center, Russia | Russia | 2015 | NCZ Krasnodar |
| 555 | 4778h29 | P. P. Lukyanenko National Grain Center, Russia | Russia | 2017 | NCZ Krasnodar |
| 556 | 4779h57 | P. P. Lukyanenko National Grain Center, Russia | Russia | 2017 | NCZ Krasnodar |
| 557 | 4812h7 | P. P. Lukyanenko National Grain Center, Russia | Russia | 2018 | NCZ Krasnodar |
| 560 | 4626h10-20-24 | P. P. Lukyanenko National Grain Center, Russia | Russia | 2017 | NCZ Krasnodar |

Table S5a. Pairwise concordance of the four *Fr-A2* markers (SNP assays tagging the *CBF-A14* copy-number haplotype) in the collection.

| **Marker 1** | **Marker 2** | **Lines called at both** | **Minor class, marker 1** | **Minor class, marker 2** | **Minor class at both** | **Concordance, %** |
| --- | --- | --- | --- | --- | --- | --- |
| S2269949 | S1077313 | 160 | 9 | 6 | 6 | 98.1 |
| S2269949 | S1862541 | 158 | 8 | 8 | 6 | 97.5 |
| S2269949 | S1298957 | 158 | 9 | 13 | 9 | 97.5 |
| S1077313 | S1862541 | 159 | 5 | 8 | 4 | 96.9 |
| S1077313 | S1298957 | 159 | 6 | 13 | 6 | 95.6 |
| S1862541 | S1298957 | 157 | 7 | 11 | 6 | 96.2 |

Table S5b. Accessions carrying the minor class at one or more of the four *Fr-A2* markers.

| **Cultivar / line** | **Markers with the minor class** | **Country** | **Origin group** |
| --- | --- | --- | --- |
| Rodur | 4 | France | foreign |
| AMA 11855t85-90 | 4 | Russia | other Russian |
| MV PENNEDUR | 4 | Hungary | foreign |
| AMA 15908ds1122-07 | 4 | Russia | other Russian |
| MV HUNDUR | 3 | Hungary | foreign |
| Leukurum 36 | 3 | Ukraine | foreign |
| 21545t | 3 | Russia | other Russian |
| 3336h6-13-162 | 3 | Russia | NCZ Krasnodar |
| Yagut | 2 | Azerbaijan | foreign |
| Donchanka | 1 | Russia | ANC Donskoy |
| Kristella | 1 | Russia | ANC Donskoy |
| Eyrena | 1 | Russia | ANC Donskoy |
| Kristall 2 | 1 | Russia | NCZ Krasnodar |
| Martondur 1 | 1 | Hungary | foreign |
| MVTD 12-99 | 1 | Hungary | foreign |

Table S6. F_2_ families: cross combination, plants genotyped before freezing, survivors and survival.

| **Family** | **Cross type** | **Plants before freezing** | **Survivors** | **Survival, %** |
| --- | --- | --- | --- | --- |
| 3320-3 | two-way | 17 | 1 | 5.9 |
| 3321-3 | three-way | 22 | 8 | 36.4 |
| 3321-5 | three-way | 19 | 7 | 36.8 |
| 3321-6 | three-way | 19 | 6 | 31.6 |
| 3321-7 | three-way | 18 | 5 | 27.8 |
| 3324-7 | three-way | 17 | 2 | 11.8 |
| 3325-3 | three-way | 20 | 8 | 40.0 |
| 3325-5 | three-way | 20 | 14 | 70.0 |

Table S7. Segregation of the *Fr-A2* markers within each F_2_ family against the expectation from the pedigree (5:2:1 for the three-way crosses, 1:2:1 for the two-way cross). P is a Monte Carlo exact chi-square test (100 000 multinomial draws). Cross combinations are given in Table S6.

| **Marker** | **Family** | **Cross type** | **n** | **Adapted homozygotes** | **Heterozygotes** | **Foreign homozygotes** | **Expected ratio** | **Chi-square** | **P (exact)** |
| --- | --- | --- | --- | --- | --- | --- | --- | --- | --- |
| S2269949 | 3320-3 | two-way | 17 | 1 | 8 | 8 | 1:2:1 | 5.82 | 0.056 |
| S2269949 | 3321-3 | three-way | 22 | 17 | 4 | 1 | 5:2:1 | 2.29 | 0.340 |
| S2269949 | 3321-5 | three-way | 18 | 16 | 0 | 2 | 5:2:1 | 6.53 | 0.038 |
| S2269949 | 3321-6 | three-way | 18 | 10 | 4 | 4 | 5:2:1 | 1.56 | 0.503 |
| S2269949 | 3321-7 | three-way | 18 | 14 | 3 | 1 | 5:2:1 | 1.87 | 0.475 |
| S2269949 | 3324-7 | three-way | 16 | 13 | 3 | 0 | 5:2:1 | 3.15 | 0.224 |
| S2269949 | 3325-3 | three-way | 20 | 14 | 4 | 2 | 5:2:1 | 0.48 | 0.804 |
| S2269949 | 3325-5 | three-way | 20 | 10 | 9 | 1 | 5:2:1 | 4.60 | 0.096 |
| S1077313 | 3320-3 | two-way | 17 | 7 | 1 | 9 | 1:2:1 | 13.71 | 0.001 |
| S1077313 | 3321-3 | three-way | 22 | 22 | 0 | 0 | 5:2:1 | 13.20 | 0.002 |
| S1077313 | 3321-5 | three-way | 17 | 17 | 0 | 0 | 5:2:1 | 10.20 | 0.007 |
| S1077313 | 3321-6 | three-way | 19 | 15 | 0 | 4 | 5:2:1 | 6.68 | 0.032 |
| S1077313 | 3321-7 | three-way | 18 | 18 | 0 | 0 | 5:2:1 | 10.80 | 0.006 |
| S1077313 | 3324-7 | three-way | 16 | 15 | 0 | 1 | 5:2:1 | 7.00 | 0.025 |
| S1077313 | 3325-3 | three-way | 20 | 18 | 0 | 2 | 5:2:1 | 7.52 | 0.023 |
| S1077313 | 3325-5 | three-way | 20 | 18 | 0 | 2 | 5:2:1 | 7.52 | 0.021 |

Table S8. Association of the polymorphic markers with frost tolerance under adjustment for origin. Values are P for the genotype term.

| **Marker** | **Unadjusted** | **+ origin group** | **+ origin group and year** | **Krasnodar program only** | **Russian material only** |
| --- | --- | --- | --- | --- | --- |
| Mor534 | 0.052 | 0.0167 | 0.041 | 0.2788 | 0.078 |
| Mor587 | 0.0484 | 0.0072 | 0.0055 | 0.1616 | 0.0118 |
| Mor866 | 0.8603 | 0.4073 | 0.406 | 0.777 | 0.3496 |
| S1051014 | 0.4416 | 0.2885 | 0.3391 | 0.5327 | 0.3537 |
| Vrn1_new | 0.0191 | 0.0658 | 0.0888 | 0.3245 | 0.3479 |

Table S9. Variance components and repeatability of the controlled freezing test.

| **Quantity** | **Value** |
| --- | --- |
| between-accession variance | 0.0357 |
| residual variance | 0.0433 |
| repeatability of a single freezing test (R) | 0.45 |
| harmonic mean number of environments per accession | 4.1 |
| repeatability of the accession mean (R) | 0.77 |
| variance explained by year and regime, % | 39.6 |
| variance explained by year, regime and accession, % | 71.2 |
| environments needed for R = 0.7 / 0.8 / 0.9 | 3 / 5 / 11 |

Table S10. Sensitivity of the marker associations to the analysis scale: P for the genotype term (likelihood-ratio test) from the primary model fitted to the angular, empirical-logit (log((y + 0.5)/(100 − y + 0.5))) and untransformed percentage scales.

| **Marker** | **Accessions** | **Angular scale** | **Empirical-logit scale** | **Untransformed percentage** |
| --- | --- | --- | --- | --- |
| S1051014 | 118 | 0.4416 | 0.3816 | 0.5560 |
| Vrn1_new | 161 | 0.0191 | 0.0116 | 0.0381 |
| Mor534 | 152 | 0.0520 | 0.0512 | 0.0566 |
| Mor587 | 156 | 0.0484 | 0.0805 | 0.0276 |
| Mor866 | 161 | 0.8603 | 0.8495 | 0.9058 |

Table S11. Mean heading date of the studied accessions (day of year), averaged over the field seasons of the Russian nursery in which each accession was observed (n = 155). These values enter model (2) and Figure 3.

| **sample_id** | **Cultivar / line** | **Years observed** | **Mean heading date, day of year** |
| --- | --- | --- | --- |
| 20 | Krupinka | 1 | 130.0 |
| 40 | Krucha | 2 | 128.5 |
| 203 | Kristall 2 | 2 | 130.5 |
| 204 | Leukurum 21 | 2 | 130.5 |
| 205 | Alena | 2 | 128.5 |
| 206 | Kermen | 1 | 129.0 |
| 207 | Uniya | 1 | 129.0 |
| 208 | Zolotko | 2 | 129.5 |
| 209 | Laska | 2 | 129.5 |
| 210 | Odari | 2 | 131.0 |
| 211 | Altana | 1 | 127.0 |
| 212 | Kordon | 2 | 130.0 |
| 213 | Dobryana | 2 | 130.0 |
| 214 | Sinora | 1 | 126.0 |
| 215 | Donchanka | 2 | 131.0 |
| 216 | Kurant | 2 | 127.5 |
| 217 | Kristella | 2 | 127.0 |
| 218 | Amazonka | 2 | 131.0 |
| 219 | Agat donskoy | 2 | 131.0 |
| 220 | Lazurit | 1 | 130.0 |
| 221 | Oniks | 1 | 130.0 |
| 222 | Diona | 2 | 132.0 |
| 223 | Kiprida | 2 | 132.0 |
| 224 | Teyya | 2 | 131.0 |
| 225 | Eyrena | 2 | 131.0 |
| 226 | Yakhont (760/10) | 1 | 129.0 |
| 227 | Yubilyarka (330/10) | 1 | 127.0 |
| 228 | Yantarina | 1 | 128.0 |
| 229 | Uslada | 1 | 128.0 |
| 230 | Zoloto Dona (993/ 12) | 1 | 129.0 |
| 231 | 796/11 | 2 | 130.5 |
| 232 | 589/13 | 1 | 128.0 |
| 233 | 737/11 | 1 | 129.0 |
| 234 | 655/13 (Solntsedar) | 1 | 130.0 |
| 235 | 533/14 | 1 | 126.0 |
| 236 | Prikumchanka | 1 | 128.0 |
| 237 | Stepnoy yantar | 1 | 127.0 |
| 238 | Zhivitsa | 2 | 130.5 |
| 239 | Bagryanitsa (Italikum) | 1 | 128.0 |
| 240 | Perlina odesskaya | 2 | 130.5 |
| 241 | Burshtyn | 2 | 129.5 |
| 242 | Kontinent | 2 | 128.5 |
| 243 | Akveduk | 2 | 130.0 |
| 244 | Andromeda | 2 | 129.0 |
| 245 | Dnepryana | 2 | 131.0 |
| 246 | Kharkovskaya 32 | 2 | 131.5 |
| 247 | Leukurum 36 | 1 | 132.0 |
| 248 | Afina | 1 | 131.0 |
| 249 | Makar | 1 | 129.0 |
| 250 | Belgorodskaya yantarnaya | 1 | 127.0 |
| 253 | Shulyndinka | 2 | 129.0 |
| 254 | Pributkova | 1 | 132.0 |
| 255 | Yagut | 2 | 132.0 |
| 256 | DF -104/85 | 2 | 127.0 |
| 257 | Pandur | 2 | 128.0 |
| 258 | Elidur | 2 | 128.5 |
| 259 | Rodur | 2 | 127.5 |
| 260 | Condur | 2 | 128.0 |
| 261 | Blindur/DF 38-86/3 | 2 | 128.0 |
| 262 | Rumyniya DF-01058-1-101 | 2 | 133.0 |
| 263 | Rumyniya DF-00091-14-002 | 2 | 132.5 |
| 264 | Martondur 1 | 2 | 131.0 |
| 265 | MVTD 12-97 | 2 | 131.0 |
| 266 | MVTD 12-99 | 2 | 130.0 |
| 267 | MVTD 15-98 | 2 | 132.0 |
| 268 | MV Makaroni | 1 | 133.0 |
| 269 | MV HUNDUR | 2 | 128.5 |
| 270 | MV PENNEDUR | 2 | 132.5 |
| 271 | Wintergold | 2 | 131.0 |
| 272 | AMA 11855t85-90 | 1 | 128.0 |
| 273 | AMA 15908ds1122-07 | 1 | 127.0 |
| 274 | 21545t | 1 | 132.0 |
| 276 | 3622h46 | 3 | 134.0 |
| 279 | 3680h62 (Belka) | 4 | 135.1 |
| 326 | 3850h69 (Tsel) | 4 | 136.7 |
| 327 | 3527h4-14-3 | 3 | 137.2 |
| 328 | 3609h36-14-4 (Bella) | 4 | 138.8 |
| 329 | 3982h42 | 3 | 137.2 |
| 330 | 3359h18-15-174 | 3 | 139.3 |
| 331 | 3896h145 | 3 | 140.3 |
| 332 | 3934h148 | 3 | 139.0 |
| 333 | 3934h155 | 3 | 139.7 |
| 334 | 4089h94 | 4 | 140.0 |
| 335 | 4135h7 | 3 | 140.0 |
| 336 | 4218h77 | 2 | 134.2 |
| 337 | 4169h75 | 1 | 144.0 |
| 338 | 4192h94 | 2 | 136.7 |
| 339 | 4192h98 | 1 | 144.0 |
| 340 | 4336h7 (Zashchita) | 3 | 140.2 |
| 341 | 4336h24 | 3 | 140.0 |
| 342 | 4337h3 | 3 | 139.8 |
| 343 | 4341h1 | 2 | 143.0 |
| 344 | 4342h23 | 1 | 144.0 |
| 345 | 3596h56-15-22 (Dara) | 3 | 140.5 |
| 346 | 3596h56-15-69 | 3 | 141.0 |
| 347 | 4306h15 | 2 | 139.0 |
| 348 | 4320h38 | 1 | 146.0 |
| 349 | 4341h68 | 1 | 145.0 |
| 350 | 4353h47 | 2 | 135.0 |
| 351 | 4383h72 | 2 | 138.5 |
| 352 | 4387h148 | 2 | 135.5 |
| 353 | 4392h17 | 2 | 135.5 |
| 354 | 4416h49 skorosp. | 2 | 134.3 |
| 355 | 4419h45 | 2 | 136.5 |
| 356 | 4424h4 | 1 | 142.0 |
| 357 | 4424h96 | 3 | 134.2 |
| 358 | 4428h110 | 2 | 136.0 |
| 359 | 4430h50 | 2 | 135.3 |
| 360 | 4432h24 | 2 | 135.0 |
| 361 | 4432h45 | 2 | 134.3 |
| 362 | 4433h52 | 2 | 134.0 |
| 363 | 4433h90 | 2 | 134.0 |
| 364 | 4466h183 | 1 | 141.0 |
| 365 | 4467h18 | 1 | 143.0 |
| 366 | 2468h10-06M-17-10 | 4 | 134.1 |
| 367 | 3336h43-17-128 | 3 | 134.0 |
| 368 | 3903h9-17-3 | 2 | 140.0 |
| 369 | 4167h48-17-12 | 3 | 140.0 |
| 370 | 4250hK-19 | 1 | 144.0 |
| 371 | 3902h3-18-31 | 3 | 135.7 |
| 372 | 3914h38-18-8 | 4 | 137.2 |
| 373 | 4477h2 | 3 | 136.3 |
| 374 | 4482h8 | 3 | 134.0 |
| 375 | 4505h38 | 2 | 137.0 |
| 376 | 4509h13 | 4 | 138.8 |
| 377 | 4517h25 | 2 | 138.5 |
| 378 | 4521h13 | 4 | 140.0 |
| 379 | 4574h8 | 3 | 134.4 |
| 380 | 4619h9 | 3 | 136.8 |
| 381 | 4619h34 | 2 | 135.5 |
| 382 | 4626h15 | 2 | 135.5 |
| 383 | 4631h14 | 4 | 137.4 |
| 384 | 4652h45 | 2 | 132.0 |
| 385 | 4681h53 | 2 | 136.0 |
| 386 | 4686h35 | 2 | 135.5 |
| 387 | 771p46-18-1 | 4 | 141.0 |
| 539 | 4519h24 | 3 | 141.0 |
| 540 | 4528h1 | 3 | 139.8 |
| 541 | 4529h25 | 3 | 139.2 |
| 542 | 4557h38 | 3 | 135.0 |
| 543 | 4626h23 | 4 | 137.0 |
| 544 | 4639h102 | 3 | 134.2 |
| 545 | 4643h29 | 3 | 136.6 |
| 546 | 4646h1 | 3 | 133.4 |
| 547 | 4675h11 | 3 | 136.5 |
| 548 | 4518h20 | 2 | 142.0 |
| 549 | 3902h3-18-3 | 3 | 136.2 |
| 550 | 4822h19 | 2 | 138.3 |
| 551 | 3408h218-18-173 | 4 | 139.8 |
| 552 | 3683h46-18-59 | 3 | 133.6 |
| 554 | 4428h48-19-11 | 1 | 140.0 |
| 555 | 4778h29 | 2 | 136.0 |
| 556 | 4779h57 | 2 | 138.3 |
| 557 | 4812h7 | 2 | 136.7 |
| 560 | 4626h10-20-24 | 2 | 136.5 |

Table S12. Frequency of the favorable (major) class at the four *Fr-A2* markers and of the major class at five control loci, by period of entry into State Variety Trials (all 161 accessions; years from Table S4). Cells give the number of accessions carrying the class / number called (frequency). The trend across individual years is a Spearman correlation between year and the class indicator. Periods were set from the distribution of years (1973–1999, n = 15; 2000–2009, n = 22; 2010–2014, n = 56; 2015–2019, n = 57; 2020–2026, n = 11).

| **Marker** | **Favorable / major class** | **1973–1999 (n = 15)** | **2000–2009 (n = 22)** | **2010–2014 (n = 56)** | **2015–2019 (n = 57)** | **2020–2026 (n = 11)** | **Spearman rho (year × class)** | **P (trend)** |
| --- | --- | --- | --- | --- | --- | --- | --- | --- |
| S2269949 | FAM (A) | 12/15 (0.80) | 19/22 (0.86) | 55/56 (0.98) | 54/57 (0.95) | 11/11 (1.00) | 0.12 | 0.1413 |
| S1077313 | HEX (A) | 13/15 (0.87) | 19/22 (0.86) | 56/56 (1.00) | 56/57 (0.98) | 11/11 (1.00) | 0.17 | 0.0304 |
| S1862541 | HEX (C) | 13/15 (0.87) | 18/22 (0.82) | 55/56 (0.98) | 54/57 (0.95) | 11/11 (1.00) | 0.12 | 0.1423 |
| S1298957 | HEX | 9/15 (0.60) | 17/22 (0.77) | 55/56 (0.98) | 54/57 (0.95) | 11/11 (1.00) | 0.26 | 0.0008 |
| S1051014 | FAM (C) | 7/15 (0.47) | 8/22 (0.36) | 19/56 (0.34) | 23/57 (0.40) | 4/11 (0.36) | −0.03 | 0.7101 |
| Vrn1_new | *vrn-A1b* (long) | 12/15 (0.80) | 20/22 (0.91) | 50/56 (0.89) | 47/57 (0.82) | 11/11 (1.00) | −0.02 | 0.8074 |
| Mor534 | G | 11/15 (0.73) | 16/22 (0.73) | 48/56 (0.86) | 35/57 (0.61) | 10/11 (0.91) | −0.08 | 0.3201 |
| Mor587 | T | 12/15 (0.80) | 15/22 (0.68) | 36/56 (0.64) | 38/57 (0.67) | 7/11 (0.64) | −0.07 | 0.3418 |
| Mor866 | A | 14/15 (0.93) | 14/22 (0.64) | 32/56 (0.57) | 34/57 (0.60) | 8/11 (0.73) | −0.07 | 0.3412 |

Figure S5. The controlled freezing program of the collection trial, shown for the −15 °C series: hardening for one day at −2 and −5 °C, cooling from −5 °C to the target at 1 °C per hour, 24 hours at the target, warming at 1 °C per hour to +5 and then to +10 °C, two days at +10 °C in the chamber, then two weeks in the glasshouse at +25 °C with 10 hours light in the first week and 14 hours light in the second.

Figure S6. Frequency of the favorable class at the four *Fr-A2* markers (solid lines) and of the major class at five control loci (gray dashed lines) by period of entry into State Variety Trials; numbers of accessions per period are given on the axis (data in Table S12).
